# Iron/Cobalt Dual‐Atom Catalyst Orchestrate Photothermal‐Chemodynamic Immunotherapy Against MRSA: Multi‐Omics Dissection in Murine and Porcine Models

**DOI:** 10.1002/advs.202516783

**Published:** 2025-12-27

**Authors:** Shihao Xu, Binge Huang, Hao Lin, Jinming Li, Zhaoxiang Lu, Qi Zhang, Jia Li, Shiping Yang, Songsong Lan, Yan Yang, Yun Feng, Xiaojun He

**Affiliations:** ^1^ Department of Ophthalmology Peking University First Hospital Beijing China; ^2^ Department of Ultrasound Medicine National Key Clinical Specialty (Wound Healing) Wenzhou Key Laboratory of Interventional Ultrasound for Intelligent Healthcare and Clinical Translation the First Affiliated Hospital of Wenzhou Medical University Wenzhou China; ^3^ School of Ophthalmology and Optometry Wenzhou Medical University Wenzhou Zhejiang China

**Keywords:** multi‐omics, single‐atom catalysts, antibacterial and antibiofilm, wound healing, anti‐infective therapy

## Abstract

Translating pathogen‐specific molecular insights into effective treatments remains a significant challenge, particularly for drug‐resistant wound infections. In this study, we develop a nitrogen‐doped iron/cobalt dual‐atom catalyst (FeCo‐N‐DAC) with high metal loading (Fe > 5.4%, Co > 4.8%) as a multifunctional platform that integrates nanozyme‐mimicking catalytic activity and photothermal therapy. FeCo‐N‐DAC mimics multiple natural enzymes to generate reactive oxygen species, disrupt bacterial biofilms, and eradicate methicillin‐resistant *Staphylococcus aureus* (MRSA) in both murine and porcine models of subcutaneous abscesses and infectious wounds, respectively. Upon near‐infrared (NIR‐II) irradiation, the material exhibits deep‐seated tissue penetration and synergistic catalytic‐photothermal effects, enabling complete biofilm clearance in otherwise recalcitrant infections. Multi‐omics analyses, including transcriptomics and proteomics, reveal that FeCo‐N‐DAC modulates immune responses and promotes tissue regeneration by reprogramming inflammation‐ and fibrosis‐related pathways. This study highlights the therapeutic potential of dual‐atom nanozymes for precision anti‐infective therapy and underscores their translational relevance in treating complex, biofilm‐associated infections.

## Graphical abstract

FeCo dual‐atom catalyst (FeCo‐N‐DAC) with ultrahigh metal loading (Fe > 5.4%, Co > 4.8%) is developed for synergistic photothermal‐chemodynamic immunotherapy. FeCo‐N‐DAC penetrates deep‐seated tissues, eradicates MRSA biofilms, and reprograms immune‐inflammatory pathways via multi‐omics‐validated mechanisms. Accelerated wound healing in murine/porcine models demonstrates its dual functionality: precise bacterial clearance and tissue regeneration. This work pioneers dual‐atom nanozymes as translational therapeutics for resistant biofilm infections.

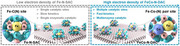

## 1 Introduction

The escalating global burden of multidrug‐resistant (MDR) bacterial infections and persistent biofilms presents a critical public health challenge, underscoring the urgent need for effective non‐antibiotic therapeutic strategies. [1, 2, 3] Conventional antibiotics, once deemed reliable, are increasingly ineffective due to the rapid evolution of bacterial resistance mechanisms and the protective extracellular matrix of biofilms. [4, 5] These biofilm structures not only serve as physical barriers but also maintain bacteria in dormant, metabolically inert states, rendering them highly resistant to both antimicrobial agents and immune clearance. [6, 7] Overcoming these multifaceted defense strategies necessitates innovative approaches capable of circumventing traditional resistance pathways and effectively eradicating biofilm‐associated infections. [8, 9, 10] Nanotechnology offers a paradigm shift in this context, offering unprecedented opportunities for precise bacterial targeting and the integration of multimodal therapeutic functions beyond the capabilities of classical antibiotics. [11, 12] Among these innovations, single‐atom catalysts (SACs) have garnered significant attention due to their maximal atom utilization, tunable electronic structures, and exceptional catalytic efficiency. [13] While SACs have been successfully employed in fields such as energy conversion and environmental remediation, their clinical translation remains hindered by poor in vivo stability, suboptimal reactive oxygen species (ROS) generation in physiological environments, and insufficient efficacy against deep‐seated biofilms. [14, 15, 16]

To address these limitations, diatomic catalysts (DACs) have emerged as promising candidates for next‐generation materials. Theoretically, the introduction of heteronuclear metal pairs within a unified coordination framework may facilitate synergistic electronic interactions between adjacent metal centers, thereby enhancing catalytic performance and structural stability. This bimetallic strategy is believed to not only maintain the high catalytic activity characteristic of single‐atom catalysts but also improve the generation of reactive oxygen species and photothermal conversion efficiency, particularly when employing metals with complementary redox properties. However, it is important to note that these potential advantages over single metal catalysts remain largely theoretical and are currently in the preliminary verification stage, lacking comprehensive validation through rigorous control experiments. Consequently, further research and experimental verification are necessary to elucidate the exact synergistic effects and performance benefits of DACs. Iron (Fe) and cobalt (Co) are especially well‐suited for constructing DACs for anti‐infective applications. [17, 18] Fe exhibits Fenton‐mimicking reactivity, a cornerstone of chemodynamic therapy (CDT), while Co provides a broader catalytic profile due to its versatile redox behavior. Both metals offer intrinsic biocompatibility and favorable electronic configurations that support photothermal activity. [19, 20, 21] Furthermore, nitrogen (N) doping enhances the functionality of DACs by increasing electrical conductivity, stabilizing high‐density metal dispersion (>5 wt.%), and modulating local electronic environments to augment enzymatic mimetic properties. These collective advantages render FeCo‐based DACs a highly promising platform for multifunctional, non‐antibiotic therapeutic strategies targeting MDR biofilm‐related infections. [22, 23, 24]

**SCHEME 1:**
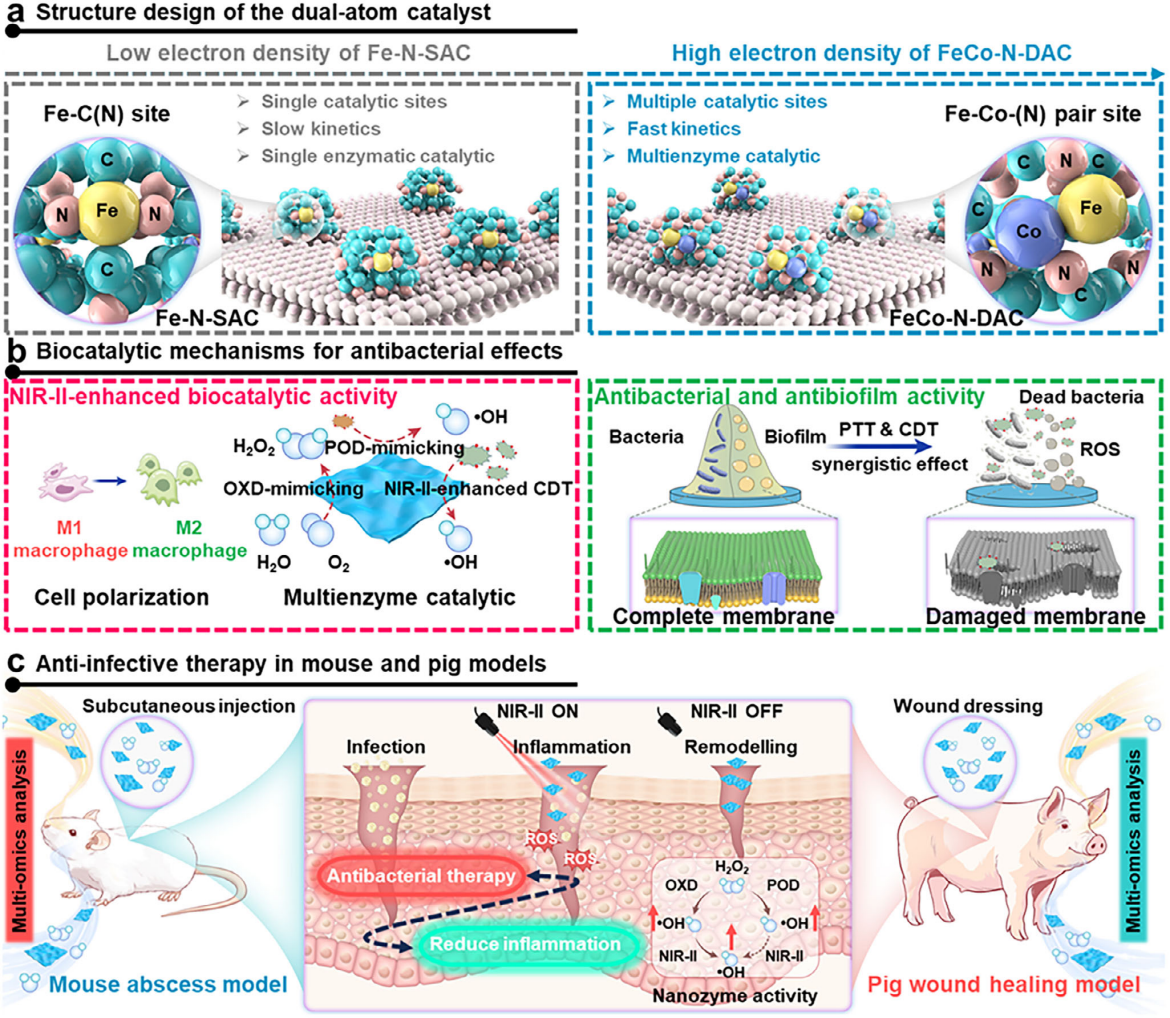
Structure, biocatalysis, and anti‐infective therapy of the FeCo‐N‐DAC nanoplatform. (a) Structural and biocatalytic advantages of FeCo‐N‐DAC compared to Fe‐N‐SAC. (b) Illustration of the biocatalytic mechanisms and bioadaptive antibacterial/antibiofilm properties of FeCo‐N‐DAC. (c) Anti‐infective therapy efficacy in mouse to pig models, demonstrating antibacterial and anti‐inflammatory applications.

In this study, we develop an N‐doped FeCo dual‐atom catalyst (FeCo‐N‐DAC) with ultrahigh metal loading (Fe > 5.4%, Co > 4.8%) that serves as a bifunctional nanozyme‐photothermal platform for treating drug‐resistant biofilm infections. The FeCo‐N‐DAC mimics multiple enzymatic activities to catalytically disrupt bacterial biofilms through localized ROS generation and enables deep‐seated tissue penetration via NIR‐II photothermal activation, synergistically enhancing catalytic efficacy. These dual mechanisms result in complete biofilm eradication, even in deep‐seated Methicillin‐resistant *Staphylococcus aureus* (MRSA)‐infected abscesses in both murine and porcine models. Mechanistic studies combining experiments and DFT simulations reveal that the atomically coordinated Fe‐Co centers drive the observed catalytic performance. Multi‐omics analyses further show that FeCo‐N‐DAC reprograms immune responses and tissue remodeling pathways, accelerating wound healing beyond bacterial clearance (Scheme 1). Collectively, these findings position DACs as a new class of precision catalytic therapeutics, where atom‐level synergy and systems‐level biological validation converge to combat biofilm‐associated infections. This work provides a rational design framework for translating dual‐atom nanotherapeutics into clinical anti‐infective applications.

## 2 Results and Discussion

### 2.1 Synthesis and Characterization of FeCo‐N‐DAC

**FIGURE 1:**
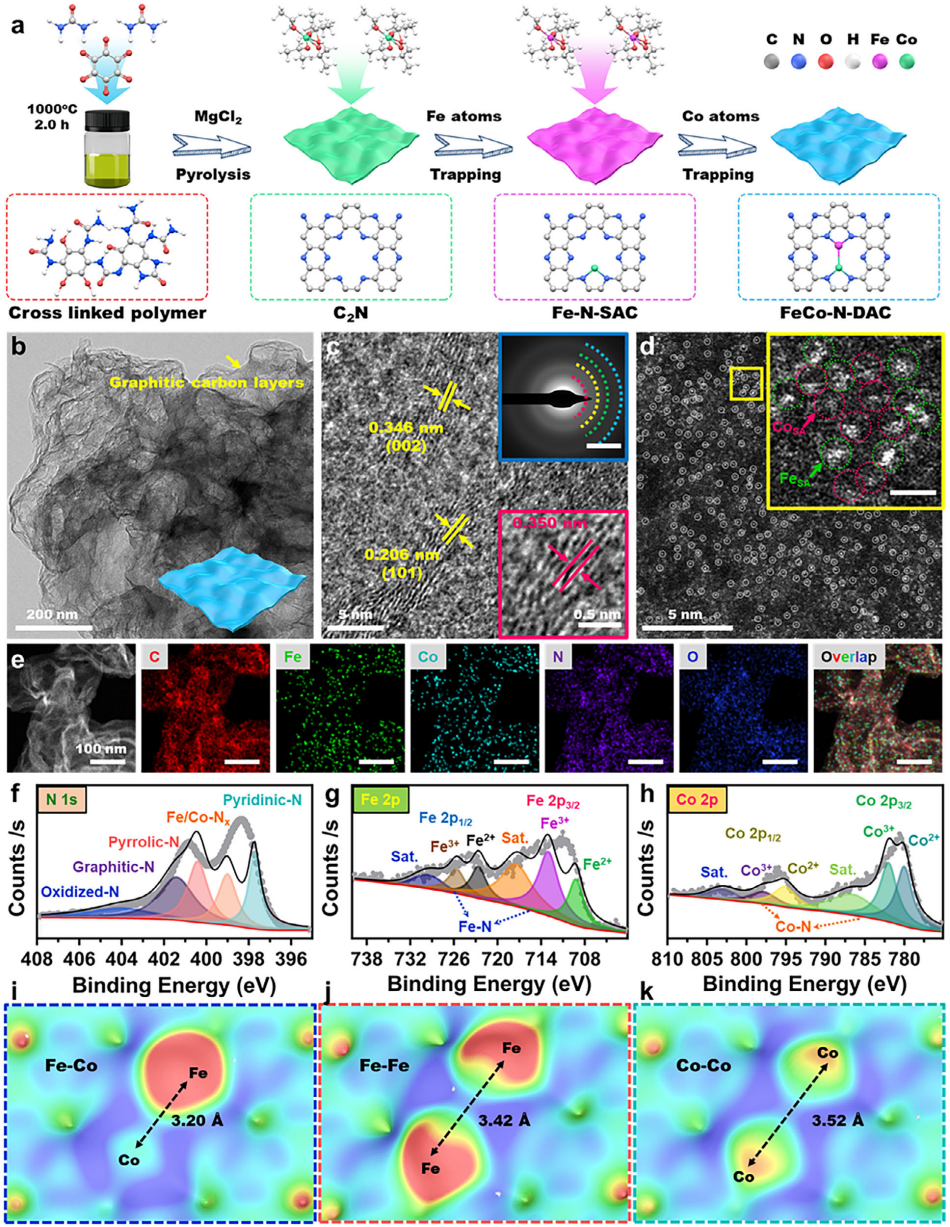
Schematic of the synthesis process and characterization of FeCo‐N‐DAC. (a) Schematic illustration of the synthetic process for FeCo‐N‐DAC. (b) TEM image of FeCo‐N‐DAC, with an inset showing the simulation structure model. (c) High‐resolution TEM (HRTEM) images of FeCo‐N‐DAC, with an inset displaying the selected area electron diffraction (SAED) pattern and a locally enlarged view. (d) HAADF‐STEM image of FeCo‐N‐DAC, with an inset showing a locally enlarged view. (e) STEM‐EDX mapping images of FeCo‐N‐DAC, showing the distributions of C (red), Fe (green), Co (cyan), N (purple), O (blue), and overlapping elements. (f) High‐resolution XPS spectrum of N 1s. (g) XPS spectrum of Fe 2p. (h) XPS spectrum of Co 2p. Intensity profiles of Fe–Co (i), Fe–Fe (j), and Co–Co (k) bimetallic pairs, respectively.

In this study, FeCo‐N‐DAC nanozymes were synthesized through a high‐temperature pyrolysis method combined with a kinetically controlled metal atom release strategy (Figure 1a). A precursor mixture consisting of PVP, urea, Fe(acac)_3_, and Co(acac)_2_ was thermally treated at 1000°C, facilitating the gradual decomposition of organometallic compounds. The slow release of Fe and Co atoms was effectively captured by an N‐doped carbon (C_2_N) matrix, resulting in atomically dispersed dual‐metal sites embedded within the N‐doped carbon framework. This method precisely regulates metal dispersion and suppresses aggregation, leading to the formation of a stable FeCo‐N‐DAC catalytic system. HAADF‐STEM, AFM, and aberration‐corrected TEM confirmed the nanosheet morphology and atomic structure (Figure 1b; Figure S1). The catalyst exhibited ultrathin layers (∼1.9 nm, Figure S2) with distinct lattice fringes at 0.206 and 0.346 nm, corresponding to the (101) plane of FeCo‐N‐DAC and the (002) plane of graphitic carbon, respectively (Figure 1c). Bright spots observed in aberration‐corrected TEM (Figure 1d; Figure S3) confirmed the presence of isolated Fe (green circles) and Co (red circles) atoms, with no metallic clusters detected. EDS mapping (Figure 1e; Figure S4) demonstrated a homogeneous distribution of C, N, O, Fe, and Co, further supported by EDS spectra (Figures S5 and S6). XRD patterns (Figure S7) displayed broad peaks at 25.0° and 44.5°, indicating partial graphitization and the absence of metallic crystallinity, consistent with the TEM results. [25, 26] Raman spectra (Figure S8) indicated an *I*_D_/*I*_G_ ratio of 0.89, suggesting that metal doping effectively modulated carbon ordering. ESR spectra (Figure S9) confirmed the presence of N‐doping, which significantly influenced graphitization and structural defects. Notably, the pyrolysis temperature was found to play a dual role: moderate temperatures facilitated graphitization, whereas excessive heating led to phase transitions and metal sublimation, thereby increasing defect density. XPS survey spectra (Figure S10a) verified the coexistence of C, N, O, Fe, and Co. The C 1s spectrum (Figure S10b) showed peaks for C═C (284.8 eV), C─N (285.4 eV), and O─C═O (289.5 eV), indicating successful N‐doping. The Fe 2p spectrum (Figure 1f) exhibited Fe^0^, Fe^2+^, and Fe^3+^ states, while the Co 2p spectrum (Figure 1g) contained signals for Co^0^, Co^2+^, and Co^3+^. The presence of Fe^0^ and Co^0^ rules out the possibility of alloy formation, which is consistent with the observed single‐atom dispersion. The N 1s spectrum (Figure 1h) revealed contributions from pyridinic‐N (398.6 eV), metal‐N (399.4 eV), graphitic‐N (401.4 eV), and oxidized‐N (403.5 eV), with pyridinic and graphitic species known to enhance electron transport. Lastly, the bimetallic pair intensity spectra (Figure 1i–k) demonstrated stronger Fe–Co interactions compared to Fe–Fe or Co–Co, suggesting the successful formation of atomically dispersed Fe–Co pairs on the N‐doped graphene substrate.

**FIGURE 2:**
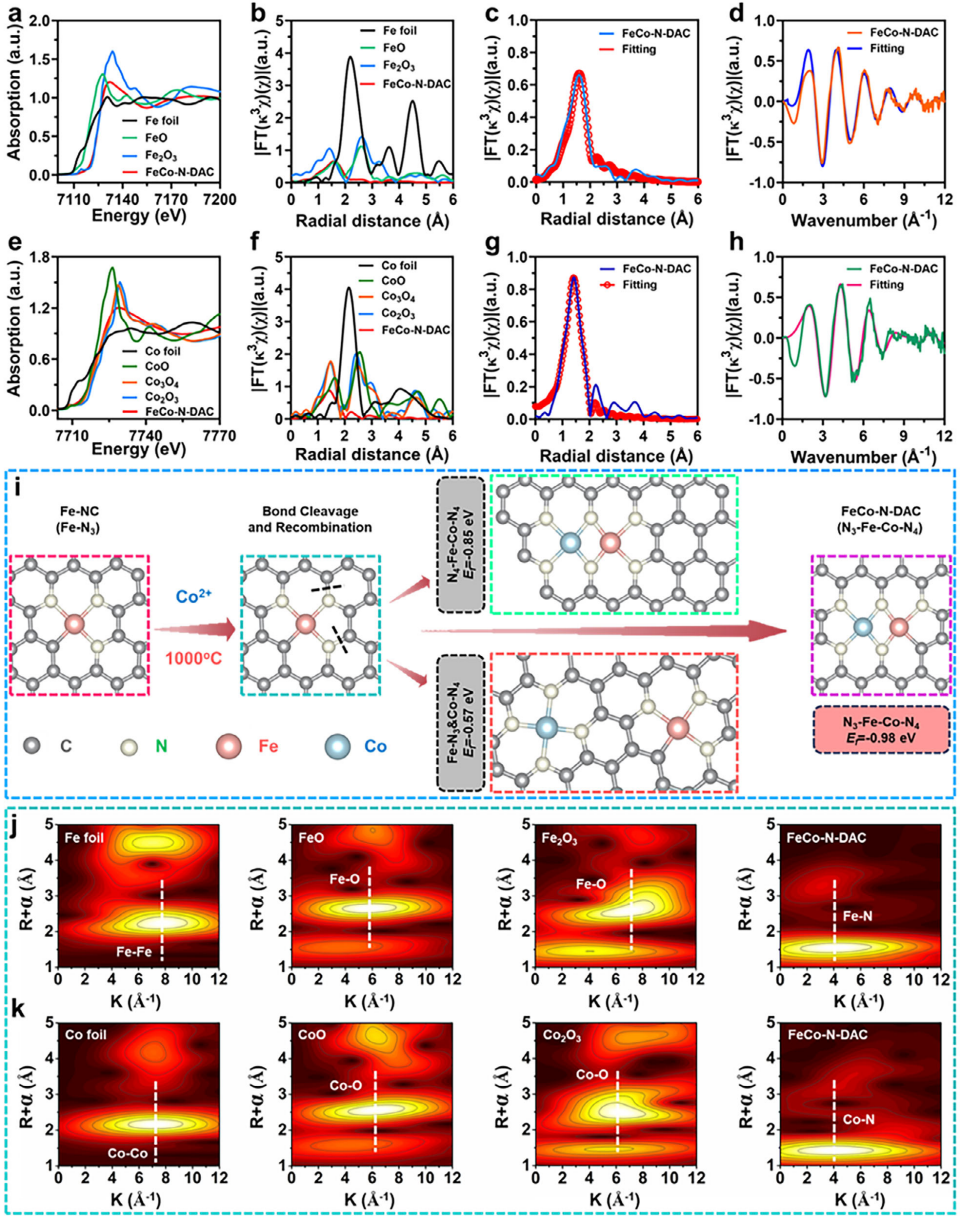
Investigation of the coordination environment of FeCo‐N‐DAC. (a) Fe *K*‐edge XANES spectra. (b) *k* ^3^‐weighted Fourier‐transformed Fe *K*‐edge EXAFS spectra, with reference materials including Fe foil, FeO, and Fe_2_O_3_. (c) Fe *K*‐edge EXAFS fitting analysis, highlighting Fe─N bonds. (d) FT‐EXAFS fitting of Fe for FeCo‐N‐DAC in R space. (e) Co‐*K*‐edge XANES spectra. (f) *k* ^3^‐weighted Fourier‐transformed Co *K*‐edge EXAFS spectra, with reference materials including Co foil, CoO, Co_2_O_3_, and Co_3_O_4_. (g) Co‐*K*‐edge EXAFS fitting analysis, highlighting Co─N bonds. (h) FT‐EXAFS fitting of Co for FeCo‐N‐DAC in R space. (i) Schematic illustrating the coordination transformation and the effective energy (Ef) of three probable configurations after the second pyrolysis. WT for the FT *k* ^3^‐weighted χ(*k*)‐the function of FeCo‐N‐DAC and reference samples at the Fe (j) and Co (k) *K*‐edges, respectively.

To elucidate the coordination environment of the active metal centers, synchrotron‐based X‐ray absorption spectroscopy (XAS) was performed. Fe *K*‐edge XANES spectra revealed a positive edge shift in FeCo‐N‐DAC compared to Fe foil (Figure 2a), indicating an elevated Fe oxidation state. The *k* ^3^‐weighted FT‐EXAFS spectra displayed prominent peaks at ∼1.6 and ∼2.2 Å (Figure 2b–d; Figure S11a,b), corresponding to first‐shell Fe–N and second‐shell Fe–Fe(Co) coordination. Similarly, Co FT‐EXAFS spectra (Figure 2e–h; Figure S11c,d) exhibited peaks at ∼1.4 and ∼2.1 Å, attributable to Co–N and Co–Co(Fe) coordination, respectively, confirming the presence of both Co‐N_X_ and Co─Fe bonds. Although Fe–Fe and Fe–Co signals cannot be fully deconvoluted due to their similar atomic numbers, the combined spectra confirm the coexistence of Fe‐N_X_ and N_X_‐Fe‐Co‐N_X_ coordination environments in FeCo‐N‐DAC. EXAFS fitting revealed a deviation from conventional Fe‐N_3_ coordination: Fe was coordinated to ∼3 N atoms (average bond length ∼2.08 Å) and Co to ∼4 N atoms (∼1.90 Å), forming a N_3_‐Fe‐Co‐N_4_ configuration. This transition is attributed to Co incorporation and the thermally driven reorganization of Fe─N and Fe─Fe bonds during high‐temperature pyrolysis. Formation energy (E_f_) calculations (Figure 2i) demonstrated that the N_3_‐Fe‐Co‐N_4_ motif had the lowest E_f_ (−0.98 eV), outperforming both N_4_‐Fe‐Co‐N_4_ (−0.85 eV) and spatially separated Fe‐N_3_ and Co‐N_4_ structures (−0.57 eV), confirming its thermodynamic favorability and structural stability. Wavelet transform (WT) analysis further supported these findings. The Fe *K*‐edge WT map (Figure 2j) revealed a dominant peak at ∼4.2 Å^−1^, characteristic of Fe–N coordination. In contrast to Fe foil, which showed a single peak at ∼7.9 Å^−1^ (indicative of metallic Fe─Fe bonding), FeCo‐N‐DAC exhibited weaker dual peaks, suggesting shorter bond lengths and reduced coordination numbers, reinforcing the presence of atomically dispersed Fe sites. WT analysis at the Co K‐edge (Figure 2k; Figure S12) also confirmed Co‐N_X_ and Fe‐Co configurations. The attenuated Fe–N and enhanced Fe–Fe(Co) signals further indicate that Co incorporation modulates Fe coordination. Importantly, no metallic Fe–Fe or Co–Co signals were detected across all XAS and HAADF‐STEM characterizations, supporting the absence of metal clustering. Collectively, these results confirm the successful construction of an atomically dispersed dual‐atom FeCo catalyst via pyrolysis and controlled metal atom release. Multi‐scale structural analyses, including HAADF‐STEM, XPS, and XAS, verify the ultrathin nanosheet morphology, N_3_‐Fe‐Co‐N_4_ coordination architecture, and the presence of multivalent Fe^2+^/Fe^3+^ and Co^2+^/Co^3+^ states. These features, tuned by N‐doping, provide a well‐defined atomic framework to support enhanced catalytic performance.

### 2.2 Theoretical Elucidation of NIR‐II Enhanced Nanozyme Mechanisms

**FIGURE 3:**
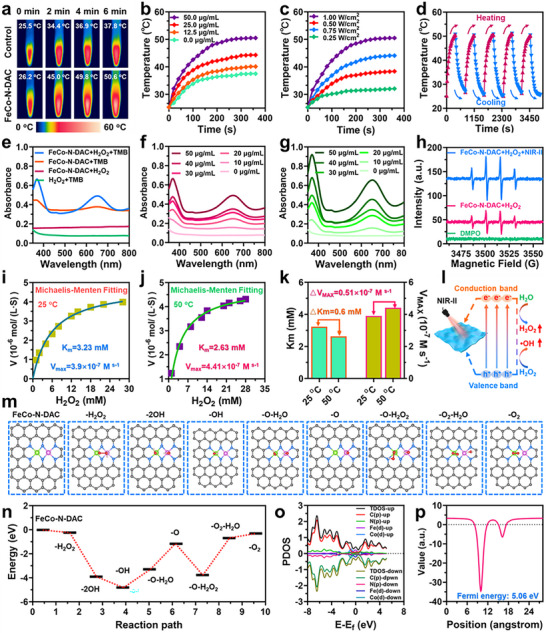
Photothermal and chemodynamic performance of FeCo‐N‐DAC. (a) Photothermal imaging of water and FeCo‐N‐DAC at the indicated concentrations (50 µg/mL). (b) Photothermal temperature elevation curve of FeCo‐N‐DAC under 1.0 W/cm^2^ laser irradiation (1064 nm) at different concentrations. (c) Photothermal temperature elevation curve of FeCo‐N‐DAC (50 µg/mL) under 1064 nm laser irradiation with varying power intensities. (d) Photothermal stability of FeCo‐N‐DAC under 1064 nm laser irradiation (1.0 W/cm^2^) during five on‐off cycles. (e) OXD‐mimicking and POD‐mimicking activities of FeCo‐N‐DAC (50 µg/mL) with TMB (1 mm) as colorimetric substrates in the presence or absence of H_2_O_2_ (1 µm) in an acidic buffer solution (pH 5.6). UV–vis absorption spectra of TMB under different concentrations of FeCo‐N‐DAC in an acidic buffer solution (pH 5.6) (f) without and (g) with 1064 nm laser irradiation (1.0 W/cm^2^). (h) The generation of •OH is illustrated by ESR spectroscopy. The Michaelis–Menten curve of FeCo‐N‐DAC (50 µg/mL) with various concentrations of H_2_O_2_ as substrates at 25°C (i) and 50°C (j) (simulating a photothermal environment). (k) Comparison of the kinetic parameters of FeCo‐N‐DAC at different temperatures. (l) Proposed mechanism of enhanced ROS generation via NIR‐II‐activated tandem catalysis of PTT and CDT based on FeCo‐N‐DAC. (m) Optimized adsorption configurations and catalytic decomposition pathways of H_2_O_2_ at Fe and Co active sites. (n) Comparative Gibbs free energy profiles for •OH generation from H_2_O_2_ decomposition. (o) Projected density of states (PDOS) for FeCo‐N‐DAC. (p) Calculated electrostatic potentials of FeCo‐N‐DAC.

The UV–vis–NIR absorption spectrum confirmed that FeCo‐N‐DAC exhibits strong absorption within the NIR‐II biological window (1000–1350 nm), supporting its application as an efficient photothermal agent (Figure S13). [27] Upon 1064 nm laser irradiation, FeCo‐N‐DAC showed concentration‐ and power‐dependent temperature elevation (Figure 3a–c), reaching 53.9°C within 5 min at 50 µg/mL and 1.0 W/cm^2^. After five heating/cooling cycles (Figure 3d), thermal stability was maintained, and a high photothermal conversion efficiency of 54.8% was calculated (Figure S14), supporting its suitability for photothermal therapy (PTT) applications. Beyond photothermal properties, FeCo‐N‐DAC exhibited dual enzyme‐mimicking functions‐oxidase‐mimicking (OXD‐mimicking) and peroxidase‐mimicking (POD‐mimicking). Optimal catalytic activity occurred at pH 5.6 (Figure S15). TMB oxidation produced a characteristic 652 nm absorption peak (Figure 3e), which increased with both catalyst concentration and reaction time (Figure 3f; Figure S16a,b). Similar trends were observed with OPD as a substrate (Figure S16c–f). Notably, NIR‐II irradiation significantly boosted POD‐mimicking activity (Figure 3g), suggesting a synergistic enhancement between photothermal effects and catalytic efficiency. Electron spin resonance (ESR) spectroscopy confirmed enhanced •OH radical generation under NIR‐II irradiation (Figure 3h), providing direct evidence of ROS amplification. Enzyme kinetics analyses using Michaelis‐Menten and Lineweaver‐Burk models (Figure 3i–l; Figure S17) revealed increased Vmax and decreased Km at 50°C (4.41 × 10^−7^ m·s^−1^, 2.63 mm) compared to 25°C (3.9 × 10^−7^ m·s^−1^, 3.23 mm), indicating improved catalytic efficiency and substrate affinity at elevated temperatures. Density functional theory (DFT) calculations further elucidated the catalytic mechanism. Optimized adsorption configurations for H_2_O_2_ intermediates (^*^OOH, ^*^O, ^*^OH) revealed localized charge distributions at Fe and Co centers (Figure 3m,n; Figure S18), with low ΔG barriers for the rate‐determining step and favorable OH desorption energy, indicating efficient reaction kinetics. The partial density of states (PDOS) near the Fermi level exhibited strong orbital hybridization among Fe, Co, C, and N (Figure 3o), and a high work function (5.06 eV; Figure 3p) suggested enhanced electron transfer capability. Collectively, these findings illustrate that single‐atom active sites impart photothermal‐catalytic synergy to FeCo‐N‐DAC by tuning its electronic structure and lowering activation barriers.

### 2.3 In Vitro Antibacterial and Antibiofilm Activities of FeCo‐N‐DAC

**FIGURE 4:**
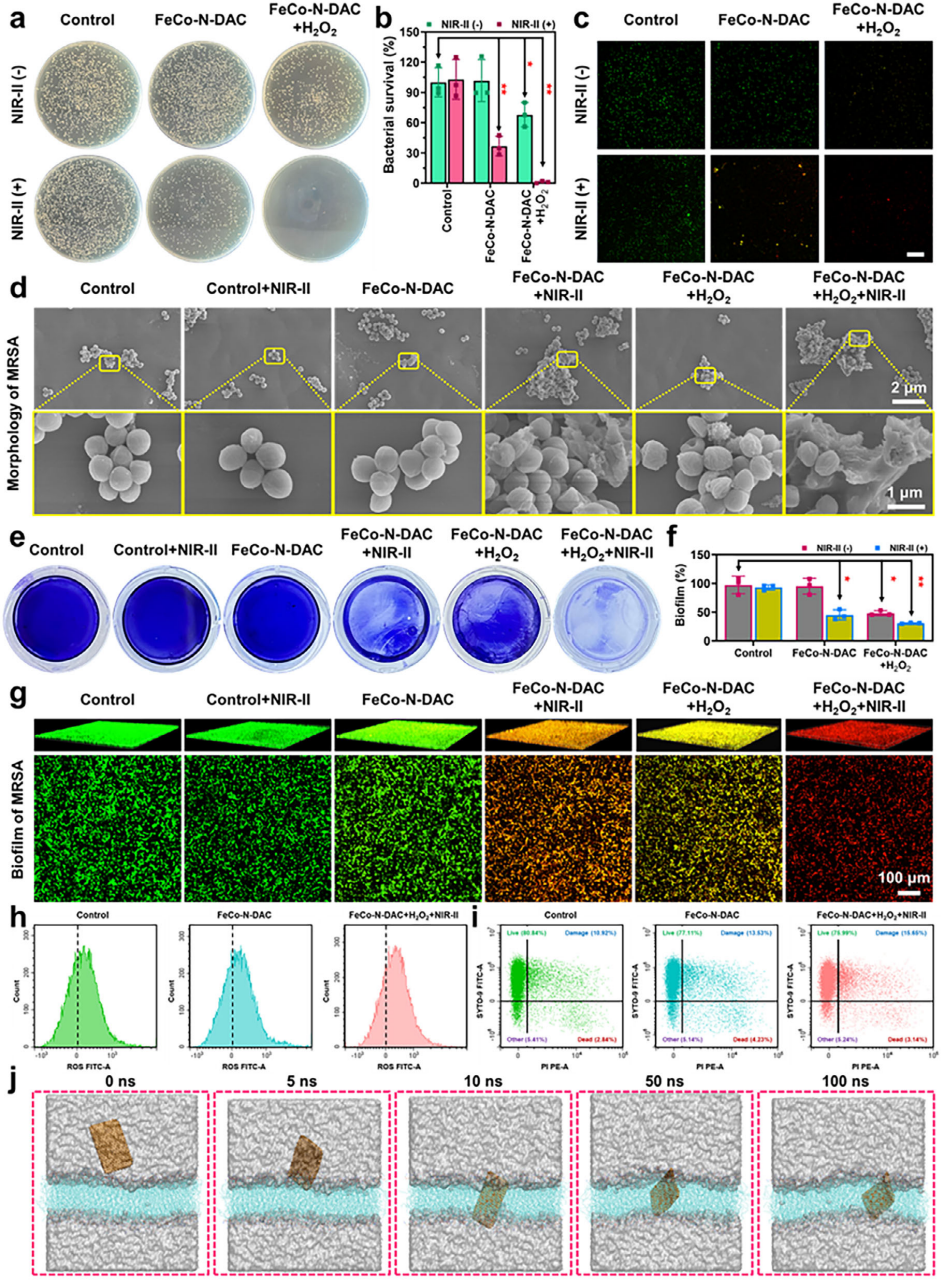
In vitro antibacterial and antibiofilm properties of FeCo‐N‐DAC. (a) Representative photographs of bacterial colonies formed on agar plates in different treatment groups, with (b) their corresponding quantitative analysis (n = 3). (c) Representative live‐dead fluorescence staining of MRSA in different treatment groups. Scale bar = 100 µm. (d) Scanning electron microscope (SEM) images showing the morphologies and structures of MRSA after different treatments. (e) Biofilm biomass of MRSA was assessed by crystal violet staining following various treatments. (f) Quantitative evaluation of the biofilm clearance ratio (n = 3). (g) 3D confocal laser scanning microscopy (CLSM) images of MRSA biofilms stained with Live/Dead dye, following incubation on Petri dishes in different treatment groups. (h) The ROS intensity and (i) Live/Damage/Dead ratio of MRSA were detected by flow cytometry under different treatment groups. (j) Conformational change of the outer membrane (OM) after FeCo‐N‐DAC interaction at 0, 5, 10, 50, and 100 ns. Data are presented as mean ± SD (n = 3), ^*^ *p* < 0.05, ^**^ *p* < 0.01, and ^***^ *p* < 0.001.

With robust biocatalytic activity, photothermal performance, and biocompatibility, FeCo‐N‐DAC emerges as a promising nanozyme platform for antibacterial and antibiofilm applications. Its two‐dimensional morphology and atomically dispersed metal sites enable FeCo‐N‐DAC to disrupt bacterial membranes and penetrate dense biofilm structures. We hypothesize that its high antibacterial efficacy is a result of the synergistic integration of CDT and PTT. [28, 29] Quantitative colony‐counting assays (Figure 4a,b) demonstrated over 99% bactericidal efficiency against MRSA under NIR‐II laser irradiation. This potent effect persisted even after accounting for the baseline antimicrobial contributions from H_2_O_2_ and the material itself (Figure S19). Live/dead fluorescence staining (Figure 4c) further confirmed this synergy: while SYTO‐9 (green) marked viable cells, propidium iodide (PI, red) labeled dead bacteria. In the FeCo‐N‐DAC + H_2_O_2_ + NIR‐II group, nearly all bacteria fluoresced red, affirming the CDT/PTT‐enhanced killing efficiency. Morphological analysis by scanning electron microscopy (Figure 4d) revealed pronounced bacterial membrane collapse and surface rupture following the combined treatment, indicating that mechanical disruption significantly contributes to bacterial eradication. To assess its antibiofilm capabilities, both crystal violet staining (Figure 4e,f) and 3D confocal laser scanning microscopy (CLSM, Figure 4g) were employed. Results confirmed a substantial reduction in biofilm biomass and viability in the synergistic treatment group, illustrating that FeCo‐N‐DAC effectively disassembled biofilm architecture and eliminated embedded pathogens. Flow cytometry analysis of intracellular ROS levels and live/damaged/dead ratios in MRSA (Figure 4h,i) revealed that upon application of FeCo‐N‐DAC+H_2_O_2_+NIR‐II, the fluorescence intensity of ROS progressively increased. Concurrently, the ratio of damaged to dead bacteria exhibited a significant increase compared to the control groups, further demonstrating the potent antibacterial mechanism of the FeCo‐N‐DAC+H_2_O_2_+NIR‐II system. Mechanistic insights were gained from molecular dynamics (MD) simulations (Figure 4j; Figures S20 and S21). These showed that FeCo‐N‐DAC adsorbs to the bacterial outer membrane (OM) and progressively penetrates it over 0–100 ns. Density distribution profiles revealed initial contact at 0 ns, with complete membrane translocation achieved by 100 ns, thus providing direct molecular‐level visualization of its membrane‐penetrating capability. In summary, the above findings demonstrate that FeCo‐N‐DAC eradicates biofilms and kills bacteria through a dual‐mode CDT/PTT effect, reinforced by its transmembrane delivery potential. This integrated therapeutic mechanism offers a compelling strategy for addressing biofilm‐associated infections.

### 2.4 FeCo‐N‐DAC Promotes Wound Healing in Mice

**FIGURE 5:**
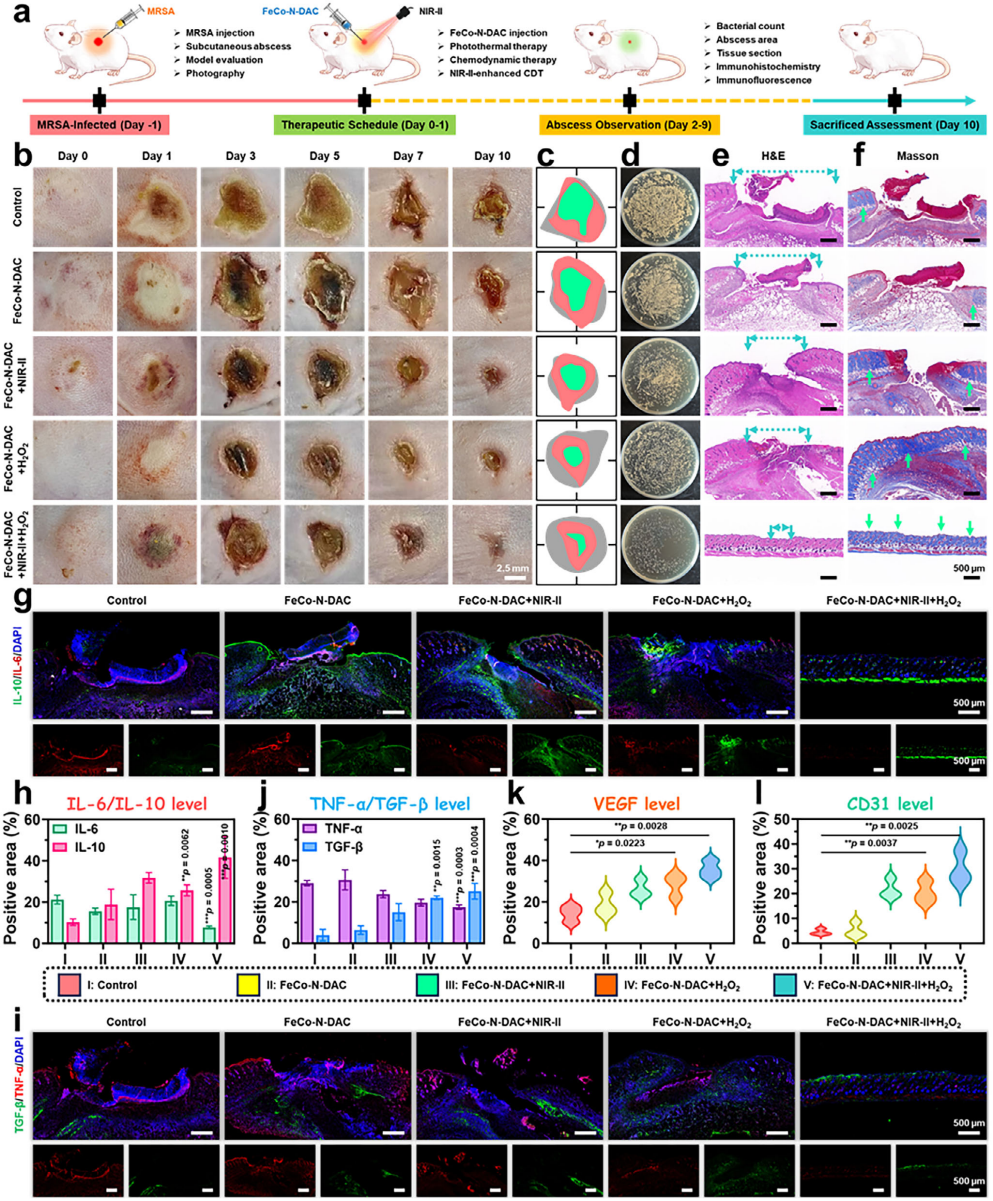
In vivo antibiofilm effects of FeCo‐N‐DAC in a subcutaneous abscess model. (a) Schematic illustration of the abscess healing procedure after treatment with FeCo‐N‐DAC dressing on subcutaneous abscesses in mice. (b) Corresponding traces of abscess healing for each treatment group in vivo. (c) Statistical analysis of the abscess area over different periods. (d) Statistical analysis of MRSA viability in different treatment groups. Histological analysis of MRSA‐infected tissues following different treatments. (e) H and E staining and (f) Masson's trichrome staining of infected tissues collected on Day 10 post‐treatment. (g) Immunofluorescence images of IL‐6 and IL‐10‐stained wound sections, and (h) the secretion levels of IL‐6 and IL‐10 in the wounds from various groups. (i) Immunofluorescence staining of macrophage biomarkers (TNF‐α and TGF‐β), and the secretion levels of (j) TNF‐α/TGF‐β, (k) VEGF, and (l) CD31 in the wounds from various groups. Data are presented as mean ± SD (n = 3), ^*^ *p* < 0.05, ^**^ *p* < 0.01, and ^***^ *p* < 0.001.

To evaluate the in vivo therapeutic potential of FeCo‐N‐DAC, including its photothermal, catalytic, and antibacterial properties, a murine subcutaneous abscess model was established via MRSA infection (Figure 5a). [30] Mice were randomly assigned to five groups: PBS, FeCo‐N‐DAC, FeCo‐N‐DAC + NIR‐II, FeCo‐N‐DAC + H_2_O_2_, and FeCo‐N‐DAC + H_2_O_2_ + NIR‐II. Over 10 days of observation, the FeCo‐N‐DAC + NIR‐II and FeCo‐N‐DAC + H_2_O_2_ + NIR‐II groups exhibited the most pronounced wound closure (Figure 5b,c), with quantitative analysis confirming the highest wound area reduction in the FeCo‐N‐DAC + H_2_O_2_ + NIR‐II group (99.2%, Figure S22). Colony counting of homogenized abscess tissues on day 10 (Figure 5d; Figure S23) revealed a dramatically reduced bacterial load in the FeCo‐N‐DAC + H_2_O_2_ + NIR‐II group (*p* < 0.001), consistent with in vitro antibacterial results. Histological analysis using H and E staining (Figure 5e; Figures S24a and S25a) showed significant scab formation in the PBS and FeCo‐N‐DAC groups, partial healing in the FeCo‐N‐DAC + H_2_O_2_ and FeCo‐N‐DAC + NIR‐II groups, and complete wound closure in the FeCo‐N‐DAC + H_2_O_2_ + NIR‐II group. Masson's trichrome staining (Figure 5f; Figure S25b) indicated denser collagen deposition in the latter group. Gram staining (Figures S24b and S25c) revealed extensive MRSA colonization in the PBS group, while only sparse bacteria were seen in the FeCo‐N‐DAC + H_2_O_2_ + NIR‐II group. Given that effective wound healing necessitates the resolution of inflammation and tissue regeneration, immunohistochemistry and immunofluorescence assays were conducted to evaluate macrophage polarization. IHC staining (Figures S26 and S27) revealed a predominance of M1 macrophages (iNOS) and a scarcity M2 macrophages (Arg1) in the PBS group, indicating persistent inflammation. In contrast, the FeCo‐N‐DAC + H_2_O_2_ + NIR‐II group demonstrated reduced iNOS levels and elevated Arg1 expression, suggesting a transition from M1‐to‐M2 macrophages. Immunofluorescence analysis (Figure 5g–j) confirmed that this treatment group downregulated pro‐inflammatory cytokines IL‐6/TNF‐α (red) while upregulating anti‐inflammatory cytokines IL‐10 and TGF‐β (green), thereby supporting effective immunomodulation. Angiogenesis was evaluated through the expression of VEGF and CD31 (Figure S28; Figure 5k,l). The FeCo‐N‐DAC + H_2_O_2_ + NIR‐II group exhibited significantly increased fluorescence for VEGF and CD31, indicating enhanced neovascularization and tissue regeneration. In summary, FeCo‐N‐DAC, under the synergistic effects of H_2_O_2_ and NIR‐II irradiation, effectively eradicates deep tissue infections, alleviates inflammation through M2 macrophage polarization, promotes collagen deposition and angiogenesis, and ultimately accelerates wound healing. These findings position FeCo‐N‐DAC as a comprehensive and potent therapeutic platform for the treatment of deep‐seated bacterial infections.

### 2.5 Multi‐Omics Reveals the Mechanism of Wound Healing in Mice

**FIGURE 6:**
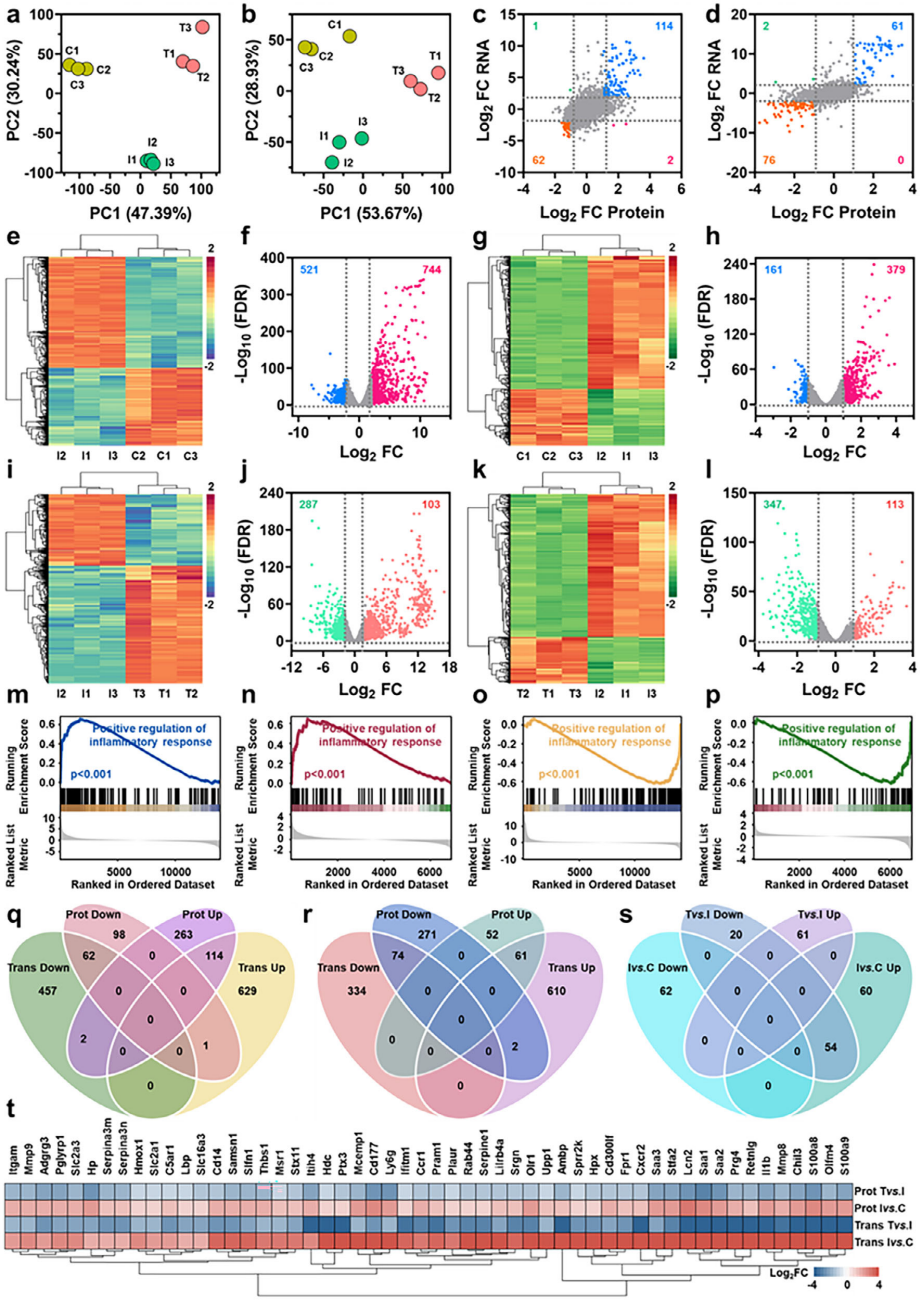
Differential expression analysis and gene set enrichment analysis (GSEA) based on transcriptomics and proteomics of mouse skin tissue before and after treatment with FeCo‐N‐DAC. Principal component analysis (PCA) plots of (a) transcriptomics and (b) proteomics samples, where yellow dots represent the control group, green dots indicate the infection group, and red dots denote the treatment group. Scatter plots illustrating the correlation between gene expression levels in proteomics and transcriptomics for (c) Ivs.C and (d) Tvs.I. Differential gene expression analysis: heatmaps and volcano plots for (e,f) transcriptomics Ivs.C, (g,h) proteomics Ivs.C, (i,j) transcriptomics Tvs.I, and (k, l) proteomics Tvs.I. Upregulated and downregulated differentially expressed genes are represented by red/pink and blue/green dots, respectively. GSEA analysis visualizing the “positive regulation of inflammatory response” biological process (BP) for (m) transcriptomics Ivs.C, (n) proteomics Ivs.C, (o) transcriptomics Tvs.I, and (p) proteomics Tvs.I. Intersection of differentially expressed genes that are upregulated and downregulated in (q) Ivs.C and (r) Tvs.I. (s) Intersection of differentially expressed gene sets showing consistent upregulation and downregulation patterns in both omics studies. (t) Log_2_ fold change (Log_2_FC) heatmap of 54 genes exhibiting consistent upregulation and downregulation patterns across both omics, with opposite expression changes observed after infection and treatment.

**FIGURE 7:**
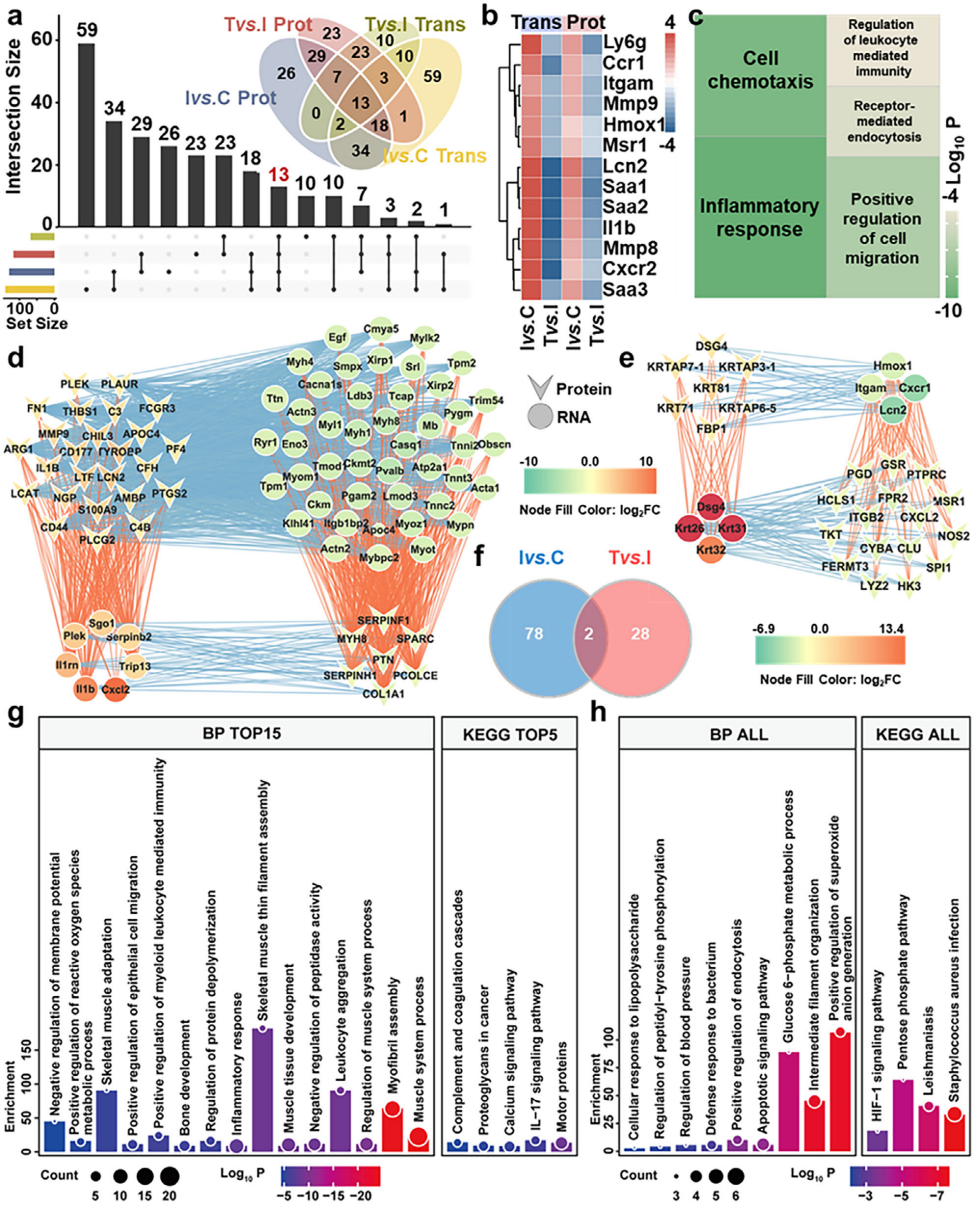
Extraction and functional enrichment of key genes in mouse skin histology. (a) Intersection of mRNA and protein in the protein interaction network diagram. (b) Log_2_FC heatmap of 13 genes present across four protein interaction networks. (c) Treemap of functional enrichment results for BP related to the 13 genes identified in four protein interaction networks. The color intensity represents the Log_10_P value of the pathway, and the square size corresponds to the number of genes enriched in each pathway. (d) Ivs.C mRNA‐protein correlation network and its central network diagram, where concave triangles represent proteins, and circles represent mRNA. The edge color indicates the correlation between mRNA and protein (red for positive, blue for negative), while the node fill color represents the Log_2_FC value. (e) mRNA‐protein correlation network and its central network diagram for Tvs.I. (f) Venn diagram depicting the intersection of key genes between Ivs.C and Tvs.I. Functional enrichment results (GO BP and KEGG) for key mRNAs and proteins in (g) Ivs.C and (h) Tvs.I.

Transcriptomic and proteomic profiling of mouse skin tissues identified 14594 mRNAs (Figure S29a) and 7072 proteins (Figure S29b), respectively. Principal component analysis (PCA) showed clear separation among the three groups in both datasets (Figure 6a,b). [31, 32] Cross‐omics comparison of log_2_ fold changes (log_2_FC) revealed high concordance in expression trends (Figure 6c,d). Upon MRSA infection, 744 mRNAs and 379 proteins were upregulated, while 521 mRNAs and 161 proteins were downregulated (Figure 6e–h). Post‐treatment with FeCo‐N‐DAC + H_2_O_2_ + NIR‐II, 103 mRNAs and 113 proteins were upregulated, while 287 mRNAs and 347 proteins were downregulated (Figure 6i–l). Gene Set Enrichment Analysis (GSEA) revealed that the biological process “positive regulation of inflammatory response” was consistently upregulated post‐infection and downregulated after treatment across both omics layers (Figure 6m–p; Figure S30a). Additionally, the “Fibrinolysis” pathway showed a similar pattern in proteomics (Figure S30b,c). Venn analysis identified 114 commonly upregulated genes post‐infection, which were enriched in the categories of “inflammatory response”, “response to external stimulus”, “neutrophil migration”, and “leishmaniasis” (Figure 6q; Figure S31a). In contrast, 62 downregulated genes were associated with “muscle system process”, “myofibril assembly”, and “motor proteins” pathways (Figure S31b). Following treatment, 61 genes were commonly upregulated (Figure 6r), enriched in “intermediate filament organization”, “keratinization”, “molting cycle”, and the “Staphylococcus aureus infection” pathway (Figure S31c). Meanwhile, 74 genes were consistently downregulated across both layers and involved in “inflammatory response”, “leukocyte chemotaxis”, and “negative regulation of response to external stimulus” (Figure S31d). Notably, 54 genes showed an infection‐induced upregulation followed by treatment‐induced downregulation (Figure 6s,t), primarily enriched in “inflammatory response”, “myeloid leukocyte migration”, “response to external stimulus”, and the “IL‐17 signaling pathway” (Figure S32, Table S1). Protein‐protein interaction (PPI) networks were constructed for all four gene sets, and core modules were extracted (Figure S33). Thirteen genes were shared across core networks (Figure 7a), all showing an infection‐upregulated and treatment‐downregulated trend (Figure 7b), mainly involved in “inflammatory response”, “cell chemotaxis”, and “cell migration” (Figure 7c). Correlation analysis of mRNA and protein expression levels identified significantly matched expression pairs (p < 0.05), from which a hub regulatory network was generated (Figure 7d,e). Among these, CXCL2 and LCN2 emerged as central regulators (Figure 7f). Further analysis identified 80 infection‐related hub genes enriched in the “muscle system process”, “myofibril assembly”, and “motor proteins” pathway (Figure 7g), and 30 treatment‐related hub genes involved in “superoxide anion generation”, “intermediate filament organization”, “glucose 6‐phosphate metabolism”, and “*Staphylococcus aureus* infection” (Figure 7h). In summary, this integrative multi‐omics analysis provides compelling molecular evidence that FeCo‐N‐DAC facilitates bacterial eradication and wound healing by reprogramming inflammatory signaling, reversing infection‐induced tissue dysfunction, and restoring skin homeostasis. The identification of CXCL2, LCN2, and other hub targets underscores the therapeutic potential of FeCo‐N‐DAC for deep infectious wounds.

### 2.6 In Vivo Wound Healing in Pigs Treated with FeCo‐N‐DAC Dressing

**FIGURE 8:**
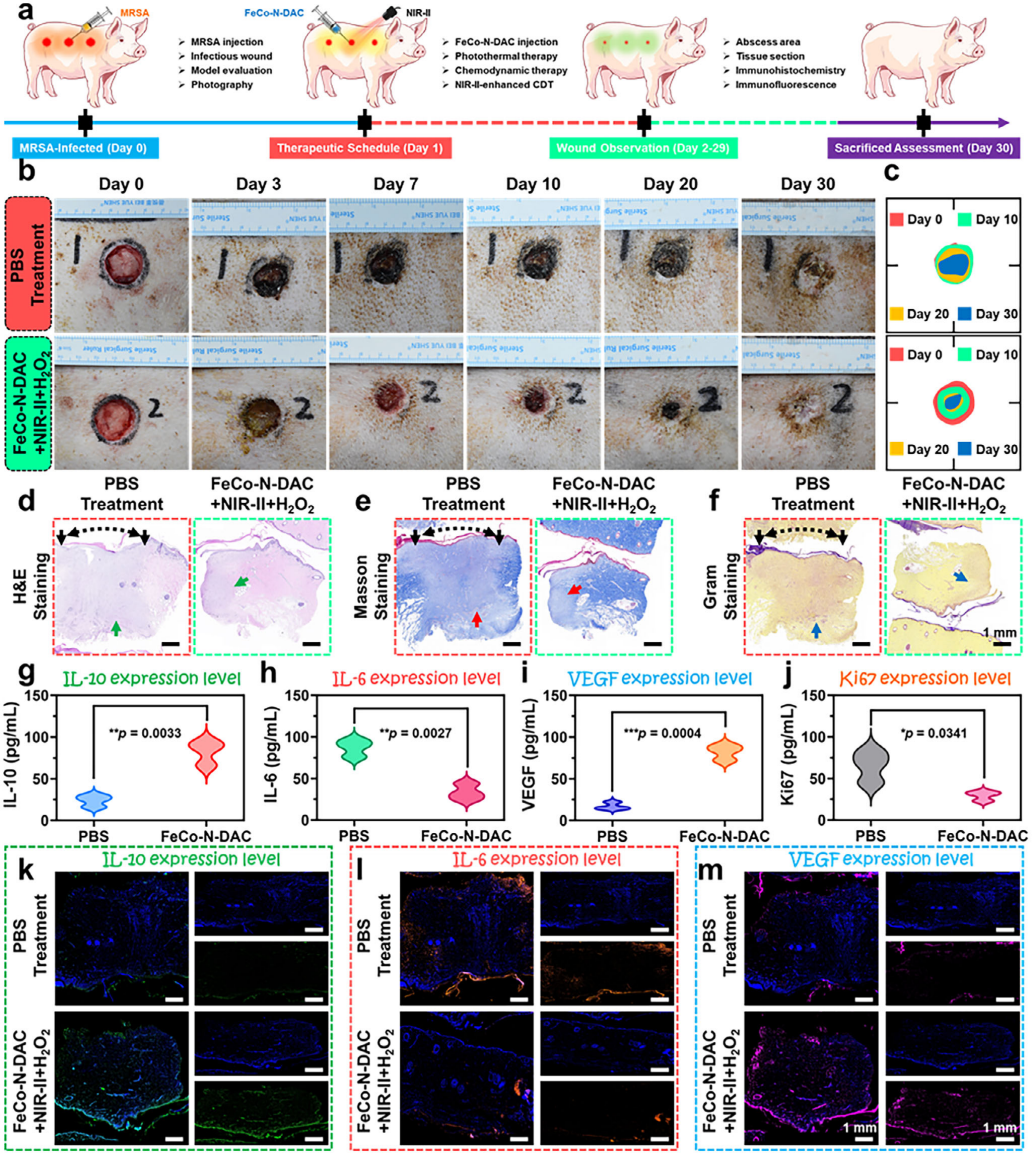
Skin infected wound healing on pigs treated by FeCo‐N‐DAC dressing. (a) A schematic illustration shows skin infected wound healing on a big white pig model, and the therapeutic procedure after treatment by FeCo‐N‐DAC dressing. (b) The images of the skin wound after different treatments as indicated. (c) Statistical analysis of the abscess area over different periods. Representative images of (d) H and E‐, (e) Masson‐, and (f) Gram‐stained wound tissues in different groups at 30 days. The expression level of IL‐10, IL‐6, VEGF, and Ki67 in pig skin tissue from various groups. Statistical data are presented as mean ± standard deviation (n = 3, ^*^ *p* < 0.05, ^**^ *p* < 0.01, and ^***^ *p* < 0.001). Representative immunofluorescence staining of (k) IL‐10, (l) IL‐6, and (m) VEGF‐stained wound sections.

To assess the preclinical therapeutic potential of FeCo‐N‐DAC, a full‐thickness skin defect model was established on the backs of Chinese large white pigs, which closely resemble human skin in anatomy and physiology (Figure 8a). [33, 34] Each group included symmetrical wounds (2.6 cm diameter, 6 mm depth) on both sides of the back. Animals were randomized into a PBS control group and a FeCo‐N‐DAC dressing treatment group (50 µg/mL, 50 µL per wound), receiving NIR‐II irradiation (1064 nm, 1.0 W/cm^2^) and 0.1 mm H_2_O_2_. All wounds were inoculated with MRSA (10^7^ CFU/mL) on day 0 and treated on day 1, then covered with gauze and bandaged. Consistent with murine results, FeCo‐N‐DAC dressing significantly accelerated wound closure in the porcine model. By day 30, wounds in the FeCo‐N‐DAC group achieved ∼90% closure versus ∼60% in the PBS group (Figure 8b,c), demonstrating its strong healing efficacy. Histological evaluation of wound edges via H and E staining (Figure 8d) revealed markedly reduced inflammation and hyperplasia in the treatment group. Notably, papillary structures re‐emerged at the dermal‐epidermal junction in treated tissues, indicating enhanced epidermal reconstruction. Masson's trichrome staining (Figure 8e) showed significantly denser collagen deposition in the FeCo‐N‐DAC group, whereas the PBS group exhibited minimal matrix formation. Importantly, signs of skin appendage regeneration, including sweat glands and anagen‐phase hair shafts, were observed in the FeCo‐N‐DAC group, features absent in the indicating superior tissue regeneration capacity. Gram staining (Figure 8f) revealed a high bacterial load in PBS‐treated wounds, while the FeCo‐N‐DAC group exhibited minimal bacterial presence, further validating its anti‐infective efficacy. To monitor inflammatory modulation and regenerative signaling, ELISA was conducted for IL‐10, IL‐6, VEGF, and Ki67 (Figure 8g–j). Compared to controls, FeCo‐N‐DAC treatment significantly increased IL‐10 and VEGF expression, while reducing pro‐inflammatory IL‐6 and proliferation marker Ki67. These changes suggest that the dressing both suppresses inflammation and promotes angiogenesis‐driven repair. Immunofluorescence staining (Figure 8k–m; Figure S34) further corroborated these results, showing elevated IL‐10 and VEGF, alongside suppressed IL‐6 and Ki67 in the treatment group. Collectively, these findings demonstrate that FeCo‐N‐DAC dressing promotes large‐area wound closure by reducing local inflammation, enhancing tissue regeneration, and accelerating the resolution of the inflammatory phase. These preclinical outcomes support its strong translational potential for treating deep and infected skin wounds.

### 2.7 Multi‐Omics Reveals the Mechanism of Wound Healing in Pigs

**FIGURE 9:**
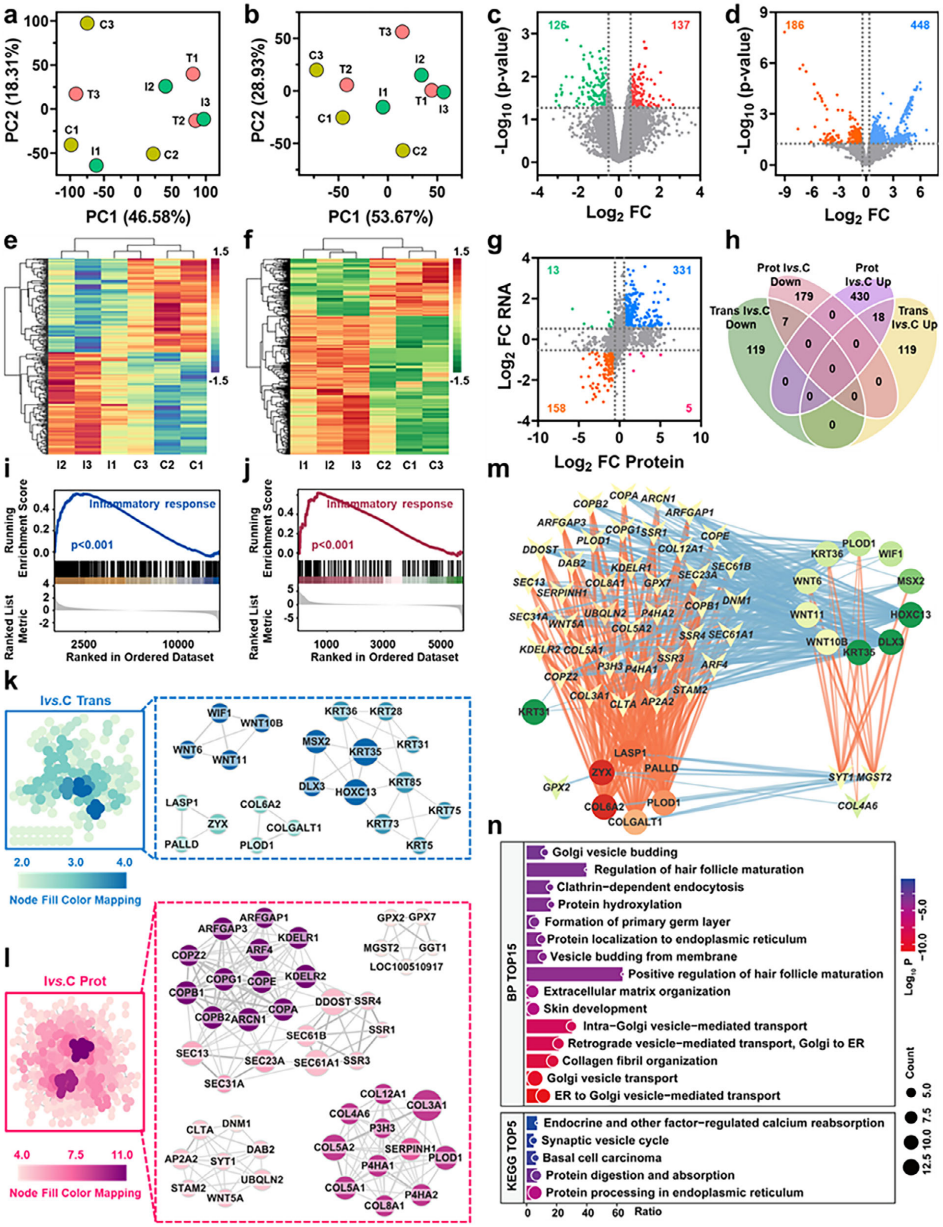
Combined transcriptomic and proteomic analysis of pig skin Ivs.C. PCA 3D plots of (a) transcriptomics and (b) proteomics samples, where yellow dots represent the control group, green dots indicate the infection group, and red dots denote the treatment group. Volcano plots of differential expression between the infection group and control group in (c) transcriptome and (d) proteome. Differential gene expression heatmaps for (e) transcriptome and (f) proteome. (g) Scatter plot showing the relationship between gene expression levels in proteomics and transcriptomics. (h) Venn diagram depicting the intersection of upregulated and downregulated genes across the two omics studies. GSEA analysis visualizes the “inflammatory response” biological process for (i) transcriptome and (j) proteome. Protein‐protein interaction networks and their central networks of differential expression mRNAs(k) and proteins(i). The color intensity represents the MCODE score, with darker colors indicating higher scores. The size of the nodes represents degree centrality (DC), with larger nodes having higher centrality. The thickness of the edges reflects the combination score, with thicker lines indicating higher scores. (m) mRNA‐protein correlation network. Concave triangles represent proteins, and circles represent mRNA. The edge color indicates the correlation (red for positive, blue for negative), while the node fill color represents the Log_2_FC value. (n) Bubble plot of functional enrichment results for key mRNAs and proteins.

**FIGURE 10:**
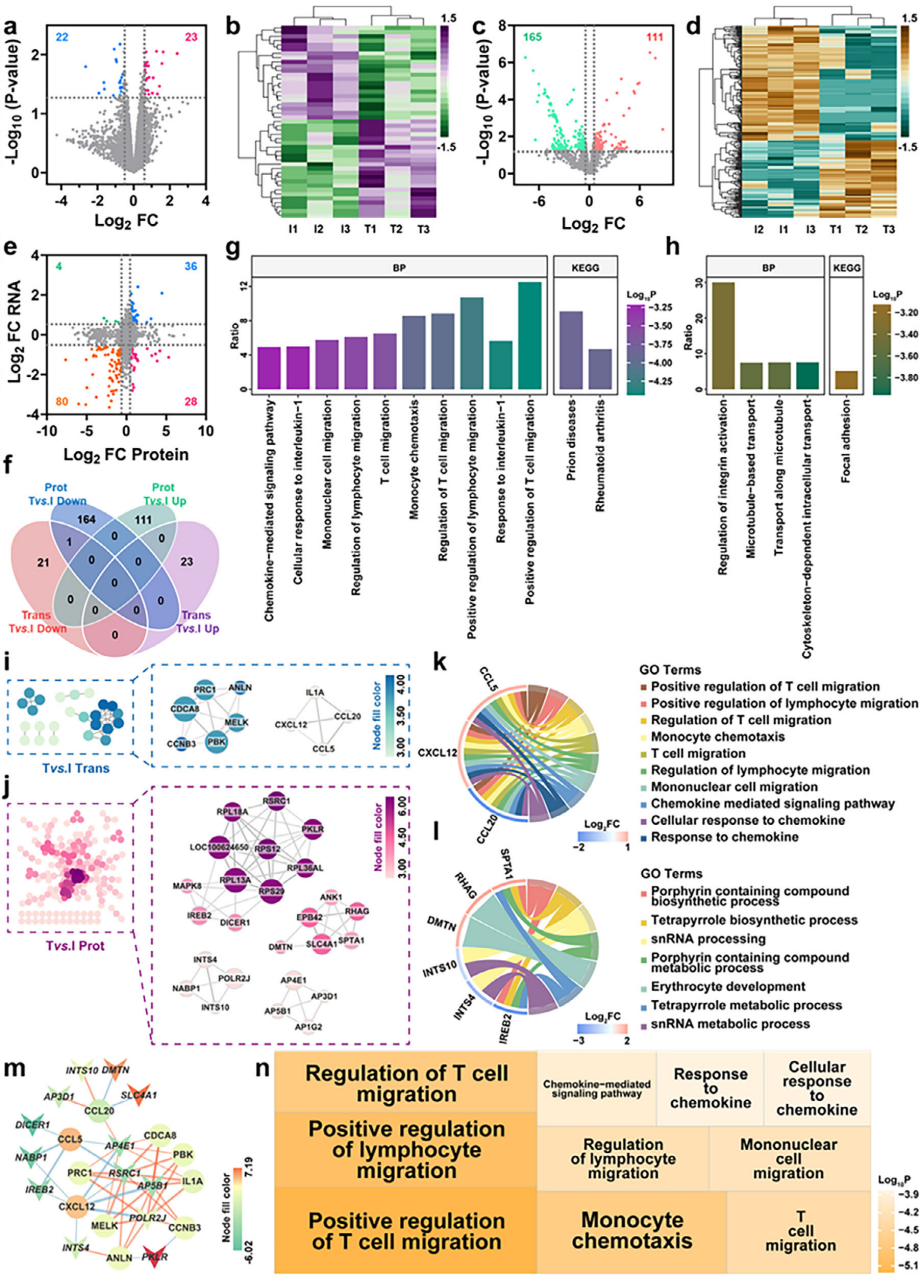
Combined transcriptomic and proteomic analysis of Tvs.I in pig skin. (a) Volcano plot of transcriptome differential expression. (b) Transcriptome differential gene expression heatmap. (c) Volcano plot of proteome differential expression. (d) Proteome differential gene expression heatmap. (e) Scatter plot showing the relationship between gene expression levels in proteomics and transcriptomics. (f) Venn diagram showing the intersection of upregulated and downregulated genes across the two omics studies. (g) Transcriptomic differential gene functional enrichment results (GO BP and KEGG). (h) Proteomic differential gene functional enrichment results (GO BP and KEGG). Protein‐protein interaction networks and their central networks of differential expression mRNAs(i) and proteins(j). The color intensity represents the MCODE score, with darker colors indicating higher scores. The size of the nodes represents degree centrality (DC), with larger nodes indicating higher centrality. The thickness of the edges represents the combination score, with thicker lines indicating higher scores. (k) Chord plot showing the relationship between the top 10 enriched mRNA BP functions and their corresponding genes. (l) Chord plot illustrating the relationship between the enriched protein BP functions and their corresponding genes. (m) mRNA‐protein correlation network. Concave triangles represent proteins, and circles represent mRNA. The edge color indicates the correlation (red for positive, blue for negative), while the node fill color represents the Log_2_FC value. (n) Treemap of functional enrichment results for key mRNAs and proteins.

Multi‐omics profiling of infected pig skin tissue identified a total of 14143 mRNAs (Figure S35a) and 5841 proteins (Figure S35b). PCA analysis of nine samples showed clear clustering and group separation at both transcriptomic and proteomic levels (Figure 9a,b). Differential expression analysis revealed that infection induced upregulation of 137 mRNAs and 448 proteins, while 126 mRNAs and 186 proteins were downregulated (Figure 9c–f). A high correlation in log_2_ fold changes (log_2_FC) across both omics datasets was observed (Figure 9g). Cross‐layer intersection identified 18 genes consistently upregulated and 7 consistently downregulated at both mRNA and protein levels post‐infection (Figure 9h; Table S2). GSEA indicated that the “inflammatory response” was significantly upregulated following infection across both omics datasets (Figure 9i,j; Figure S36). PPI network construction and core module extraction (Figure 9k,l) revealed key regulators enriched in biological processes, including “ER to Golgi vesicle‐mediated transport,” “collagen fibril organization,” and the KEGG pathway “protein processing in endoplasmic reticulum” (Figure S37; Figure 9n). A correlation network of mRNA‐protein expression (p < 0.05) highlighted 17 core mRNAs and 43 core proteins (Figure 9m), with PLOD1 identified as a shared hub gene involved in extracellular matrix biosynthesis and remodeling. Following FeCo‐N‐DAC treatment, 23 mRNAs were upregulated and 22 were downregulated (Figure 10a,b), while 111 proteins were upregulated and 165 were downregulated (Figure 10c,d). Consistent log_2_FC trends across omics layers were observed again (Figure 10e). Among the overlapping differentially expressed genes, RHOD was consistently downregulated at both the mRNA and protein levels post‐treatment (Figure 10f; Table S3), suggesting its potential as a therapeutic response marker. GO enrichment analysis revealed that the 45 differentially expressed mRNAs were primarily involved in immune‐related processes such as “positive regulation of T cell migration,” “response to interleukin‐1,” and the “rheumatoid arthritis” pathway (Figure 10g). Concurrently, the 276 differentially expressed proteins were enriched in “extracellular matrix organization,” “supramolecular fiber organization,” and “protein processing in endoplasmic reticulum” (Figure 10h). PPI networks and extracted core modules (Figure 10i,j) further emphasized immune regulation and matrix remodeling as key processes. Specifically, core mRNA networks were enriched in “T cell migration” and immune activation (Figure 10k), while core protein networks were associated with “porphyrin‐containing compound biosynthesis” (Figure 10l). Cross‐omics correlation analysis (Figure 10m) identified key hub regulators involved in “positive regulation of T cell and lymphocyte migration” (Figure 10n). In conclusion, the integrative multi‐omics analysis of pig skin tissue revealed critical transcriptomic and proteomic alterations following MRSA infection and subsequent FeCo‐N‐DAC treatment. Inflammation, immune cell recruitment, and extracellular matrix remodeling emerged as central therapeutic targets. Hub genes such as PLOD1 and RHOD provide molecular insight into the healing process, reinforcing the mechanistic basis for FeCo‐N‐DAC's immunomodulatory and regenerative efficacy in large‐animal wound healing models.

### 2.8 Biosafety Assessment of FeCo‐N‐DAC

To comprehensively evaluate the biosafety of FeCo‐N‐DAC and support its future clinical translation for anti‐infective therapies, systematic toxicity assessments were conducted both in vitro and in vivo. [35, 36] For the in vitro evaluation, L929 mouse fibroblasts were utilized to assess cytotoxicity via the CCK‐8 assay after a 24‐h exposure to FeCo‐N‐DAC at concentrations ranging from 0 to 100 µg/mL. Cell viability remained above 80% even at the highest concentration, indicating excellent biocompatibility and negligible cytotoxicity (Figure S38). In vivo, biosafety was examined through serum biochemical and hematological analyses. Liver and kidney function indicators, including alanine aminotransferase (ALT), aspartate aminotransferase (AST), blood urea nitrogen (BUN), and creatine kinase (CK)‐all remained within physiological ranges (Figure S39a–d), suggesting no evident hepatic or renal toxicity. Hematological indices such as white blood cells (WBC), monocytes (Mon), lymphocytes (Lymph), granulocytes (Gran), mean corpuscular volume (MCV), platelet count (PLT), and gamma‐glutamyl transferase (α‐GT) also showed no pathological deviations (Figure S39e–k), confirming hematological stability. To assess hemocompatibility, a hemolysis assay was performed. Red blood cells exposed to FeCo‐N‐DAC at 200 µg/mL for 6 hours exhibited no significant hemolysis (Figure S40), further validating its excellent blood compatibility. In summary, across multiple safety parameters‐including cellular viability, serum biochemistry, hematology, and hemolysis‐FeCo‐N‐DAC demonstrated minimal toxicity and high biocompatibility both in vitro and in vivo. Importantly, no adverse effects were observed even under photothermal (PTT) and chemodynamic (CDT) activation conditions. These findings strongly support the safety profile of FeCo‐N‐DAC and lay a solid foundation for its clinical development in treating deep‐seated infections and related complex pathologies.

## 3 Conclusion

In this study, we developed an N‐doped FeCo dual‐atom catalyst (FeCo‐N‐DAC) that features high metal loading and atomically dispersed bimetallic sites, serving as a robust platform for synergistic photothermal‐chemodynamic antibacterial therapy. Advanced structural characterizations and DFT simulations revealed a unique N_3_‐Fe‐Co‐N_4_ coordination architecture, which endows the catalyst with an optimized electronic structure for enhanced catalytic efficiency and NIR‐II‐responsive photothermal conversion. The FeCo‐N‐DAC exhibits potent enzyme‐mimetic activity, enabling efficient ROS generation under complex physiological conditions. Combined with strong NIR‐II photothermal effects, the catalyst effectively disrupts MRSA biofilms and penetrates bacterial membranes via a transmembrane delivery mechanism. In vivo, FeCo‐N‐DAC eradicates biofilm‐associated infections and markedly accelerates wound healing in both murine and porcine abscess models by promoting M2 macrophage polarization, enhancing angiogenesis, and suppressing inflammatory cytokines. Multi‐omics profiling further reveals that FeCo‐N‐DAC reprograms immune‐inflammatory networks and restores tissue homeostasis by modulating key molecular targets such as CXCL2, LCN2, PLOD1, and RHOD. Importantly, comprehensive biosafety assessments confirm excellent biocompatibility at both cellular and systemic levels. These findings position FeCo‐N‐DAC as a next‐generation dual‐atom nanozyme with multifunctional antibacterial capabilities and precise immunomodulatory effects, offering a promising strategy for treating biofilm‐related infections and promoting tissue regeneration.

## 4 Experimental Section

### Synthesis of FeCo‐N‐DAC

Cyclohexanone and urea were mixed in a molar ratio of 1:5 and thoroughly ground. The mixture was slowly heated at 95°C for 1 h. The resulting crosslinked product was ground with anhydrous MgCl_2_ in a mass ratio of 1:3.5. The mixed powder was then calcined in a tubular furnace under nitrogen flow at 1000°C for 2 h with a heating rate of 3°C min^−1^. After cooling to room temperature, the sample was washed sequentially with 0.5 m sulfuric acid, deionized water, and ethanol. The product was dried overnight in a vacuum oven at 60°C to obtain C_2_N. Subsequently, 90 mg of the C_2_N product was mixed with 5 mg of iron (III) acetylacetonate by grinding. The mixture was then heated under nitrogen in a tubular furnace at 1000°C for 2 h (heating rate: 3°C min^−1^) to obtain Fe‐N‐SAC. Finally, the Fe‐N‐SAC was mixed with a methanol solution containing 8 mg cobalt (II) acetylacetonate and again annealed at 1000°C under nitrogen for 2 h, yielding FeCo‐N‐DAC for direct use without further treatment.

### Dual Enzyme‐Mimicking Activity of FeCo‐N‐DAC

To evaluate oxidase‐mimicking activity, TMB (1 mm) was used as the substrate under acidic conditions (pH 5.6). [37] Two groups were set: control (room temperature) and experimental (with 1.0 W/cm^2^ NIR‐II irradiation). FeCo‐N‐DAC (50 µg/mL) was mixed with TMB and reacted under the respective conditions. UV–vis–NIR absorption spectra were measured after 60 s. POD‐mimicking activity: TMB (1.0 mm) and H_2_O_2_ (1.0 mm) were used as substrates to evaluate peroxidase‐mimicking activity at varying FeCo‐N‐DAC concentrations (0, 10, 20, 30, 40, and 50 µg/mL). Two groups (control and NIR‐II irradiated) were scanned at 0, 20, 40, and 60 seconds using full‐wavelength UV–vis spectroscopy.

### Mouse Abscess Model and Treatment

Female BALB/c mice (5‐6 weeks old) were used to establish a subcutaneous abscess model. MRSA suspension (1 × 10^7^ CFU/mL) was injected subcutaneously (50 µL) on the back of each mouse. [38, 39] After 24 h, mice were divided into five groups (n = 6): 1) PBS; 2) FeCo‐N‐DAC; 3) FeCo‐N‐DAC + NIR‐II; 4) FeCo‐N‐DAC + H_2_O_2_; 5) FeCo‐N‐DAC + H_2_O_2_ + NIR‐II. Grous (2), (3), and (5) received 50 µL of FeCo‐N‐DAC (100 µg/mL) via local injection. Groups (3) and (5) were irradiated with NIR‐II (1064 nm, 1.0 W/cm^2^, 6 min), with thermal imaging used to monitor temperature. Groups (4) and (5) received H_2_O_2_ (0.1 mm). Abscesses were photographed on days 0, 1, 3, 5, 7, and 10. On day 10, mice were euthanized for tissue analysis. Skin samples were homogenized for bacteria counting, and major organs were collected for histology (H&E, immunofluorescence, and Masson staining).

### Porcine Wound Infection Model

Four healthy, large white pigs were anesthetized, shaved, sterilized, and draped. On each pig's back, five circular full‐thickness skin wounds (diameter ∼2.0 cm, depth ∼6 mm) were symmetrically created on each side, totaling 10 wounds per pig, spaced 6–8 cm apart. After photographing initial wounds, pigs were randomly divided into control and treatment groups (n = 3 pigs/group). Each wound was inoculated with 300–500 µL MRSA (1 × 10⁷ CFU/mL). The control group received PBS, while the treatment group received FeCo‐N‐DAC (50 µg/mL), H_2_O_2_ (0.1 mm), and NIR‐II irradiation (1.0 W/cm^2^). Wound healing was monitored via photographs on days 1, 3, 5, 7, 10, 15, 20, 25, and 30. The wound area was quantified using ImageJ. On day 30, pigs were sacrificed, and tissues were fixed in 4% paraformaldehyde for paraffin embedding, H and E staining, immunohistochemistry, and immunofluorescence staining.

### Ethical Statement

All animal procedures were performed following the Guidelines for Care and Use of Laboratory Animals of Wenzhou Medical University and approved by the Animal Ethics Committee of SYXK‐2021‐0020. Experiments on wound healing in pigs were conducted following relevant guidelines and regulations. The experimental protocol received approval from the Institutional Animal Ethics Committee (Approval Number: SYXK‐2023‐0020). Pigs were housed under strictly controlled environmental conditions: the temperature was maintained between 20°C and 26°C, relative humidity was kept at 40%–70%, and a 12‐h light/dark cycle was provided. The animals were fed a standardized maintenance diet (manufactured by XieTong Pharmaceutical Bioengineering Feed Co., Ltd.) specifically formulated for laboratory animals, adhering to the nutritional specifications outlined in the Chinese standard Q/320115 XT06‐2022 (Nutritional Components of Compound Feed for Laboratory Animals). All procedures were designed to minimize discomfort and stress to the animals.

### Statistical Analysis

All experiments were performed in triplicate. Data are presented as mean ± standard deviation (SD, n = 3). Statistical significance was assessed using one‐way ANOVA and/or Student's t‐test. P‐values were considered significant as follows: ^*^ *P* < 0.05, ^**^ *P* < 0.01, and ^***^ *P* < 0.001.

## Conflicts of Interest

The authors declare no conflict of interest.

## Supporting information




**Supporting File 1**: advs73555‐sup‐0001‐SuppMat.docx

## Data Availability

The data that support the findings of this study are available from the corresponding author upon reasonable request.
