# Supplementary material for: Iron/Cobalt Dual‐Atom Catalyst Orchestrate Photothermal‐Chemodynamic Immunotherapy Against MRSA: Multi‐Omics Dissection in Murine and Porcine Models
Source: Adv Sci (Weinh). 2025 Dec 27;13(14):e16783. doi: 10.1002/advs.202516783 (PMC12970195; doi:10.1002/advs.202516783)
Supplement: Supplementary file 1 — Supporting File 1: advs73555‐sup‐0001‐SuppMat.docx [file ADVS-13-e16783-s001.docx]

Supporting Information

Iron/Cobalt Dual-Atom Catalyst Orchestrate Photothermal-Chemodynamic Immunotherapy against MRSA: Multi-Omics Dissection in Murine and Porcine Models

Shihao Xu, Binge Huang, Hao Lin, Jinming Li, Zhaoxiang Lu, Qi Zhang, Jia Li, Shiping Yang, Songsong Lan, Yan Yang,* Yun Feng,* and Xiaojun He*

H. Lin, Z. Lu, J. Li, Y. Yang, Y. Feng, X. He

Department of Ophthalmology, Peking University First Hospital, Beijing, 100034, China

E-mail: [21394@pkufh.com](mailto:21394@pkufh.com) (X. He), [fengyun@bjmu.edc.cn](mailto:fengyun@bjmu.edc.cn) (Y. Feng), [21409@pkufh.com](mailto:21409@pkufh.com)

(Y. Yang)

S. Xu, Q. Zhang, J. Li, S. Yang, S. Lan

Department of Ultrasound Medicine, National Key Clinical Specialty (Wound Healing), Wenzhou Key Laboratory of Interventional Ultrasound for Intelligent Healthcare and Clinical Translation, the First Affiliated Hospital of Wenzhou Medical University, Wenzhou 325000, China

B. Huang, X. He

School of Ophthalmology & Optometry, Wenzhou Medical University, Wenzhou, Zhejiang 325035, China

**1. Experimental Section**

**1.1 Materials and Equipment**

All chemicals used in this study were of analytical grade and used without further purification. Cyclohexanone, urea, magnesium chloride, o-phenylenediamine (OPD), sulfuric acid, 3,3',5,5'-tetramethylbenzidine (TMB), methylene blue (MB), iron (III) acetylacetonate, and cobalt (II) acetylacetonate were purchased from Aladdin Reagent Co., Ltd. Tryptic Soy Broth (TSB) and agar were obtained from Solarbio Life Sciences, Beijing. The main instruments used in the experiments include: Transmission Electron Microscope (TEM): FEI Talos F200S (200 kV); Scanning Electron Microscope (SEM): Zeiss Sigma 500 (equipped with XFlash 6130 detector); X-ray Diffractometer (XRD): Rigaku SmartLab; X-ray Photoelectron Spectroscopy (XPS): Thermo Scientific ESCALAB Nexsa G2; Electron Paramagnetic Resonance (EPR): Bruker A300; Atomic Force Microscope (AFM): Bruker Dimension Edge; Raman Spectrometer: HORIBA HR Evolution; Spherical aberration-corrected TEM: Thermo Scientific Titan Cubed Themis G2 300; UV-vis-NIR Spectrophotometer: PerkinElmer Lambda 950. X-ray Absorption Spectroscopy (XAS) data for FeCo-N-DAC, including Fe *K*-edge (7112 eV) and Co *K*-edge (7709 eV) X-ray Absorption Near Edge Structure (XANES) and Extended X-ray Absorption Fine Structure (EXAFS), were collected at the Singapore Synchrotron Light Source (SSLS). A pair of channel-cut Si (111) crystals was used in the monochromator. All XANES data were collected in transmission mode. Fe foil, FeO, and Fe_2_O_3_ were used as references for Fe *K*-edge measurements, and Co foil, CoO, Co_2_O_3_, and Co_3_O_4_ were used for Co *K*-edge measurements.

**1.2 Photothermal Effect of FeCo-N-DAC**

Concentration- and power-dependent testing: FeCo-N-DAC was dispersed in the solvent at concentrations of 0, 12.5, 25, and 50 µg/mL. Each sample was irradiated with an NIR-II laser at different power densities (0.25, 0.5, 0.75, and 1.0 W/cm^2^) for 6 min. Real-time temperature was recorded every 20 sec, and thermal images were captured using an infrared camera. Photothermal stability testing: FeCo-N-DAC was irradiated under NIR-II laser at 1.0 W/cm^2^ for 6 min, followed by a 7 min rest at 25^o^C. This heating-cooling cycle was repeated 5 times, with continuous temperature monitoring.

**1.3 Enzyme Kinetics Analysis**

To determine kinetic parameters using H_2_O_2_ as a substrate, TMB (1.0 mM) and FeCo-N-DAC (50 µg/mL) concentrations were fixed while varying H_2_O_2_ concentrations. Two groups (25^o^C and NIR-II irradiated at 1.0 W/cm^2^) were tested. After 1 min of reaction, absorbance at 652 nm was measured. Michaelis-Menten fitting was used to calculate K_m_ and V_max_.

**1.4 Theoretical Calculations**

Calculations were performed using the CP2K software package within the DFT framework, utilizing the Gaussian and plane wave (GPW) hybrid basis set. The exchange-correlation function used was PBE under the generalized gradient approximation (GGA). Core electrons were treated using Goedecker-Teter-Hutter (GTH) pseudopotentials, and valence electrons used the DZVP-MOLOPT-SR-GTH basis set. The plane wave energy cutoff was set at 600 Ry. Convergence criteria were maximum atomic force < 4.5 × 10^-4^ Hartree/Bohr and energy change < 1 × 10^-5^ eV between iterations. Monkhorst-Pack K-point grid of 3 × 3 × 1 was used.

**1.5 *In Vitro* Antibacterial Assays of FeCo-N-DAC**

Methicillin-resistant *Staphylococcus aureus* (MRSA) was cultured overnight at 37^o^C in a shaking incubator. Bacterial suspensions in the logarithmic growth phase were used. The experiment consisted of 6 groups: (1) MRSA group: only MRSA (1×10^7^ CFU/mL); (2) NIR-II: MRSA + NIR-II irradiation; (3) FeCo-N-DAC: MRSA + FeCo-N-DAC (50 µg/mL); (4) FeCo-N-DAC/H_2_O_2_: MRSA + FeCo-N-DAC + H_2_O_2_ (0.1 mM); (5) FeCo-N-DAC/NIR-II: MRSA + FeCo-N-DAC + NIR-II; (6) FeCo-N-DAC/H_2_O_2_/NIR-II: MRSA + FeCo-N-DAC + H_2_O_2_ + NIR-II. All groups were incubated at room temperature for 1 h. Groups (2), (5), and (6) were irradiated with an NIR-II laser (1.0 W/cm^2^, 6 min). After treatment, bacterial suspensions were diluted 625-fold and plated on TSB agar. Plates were incubated at 37^o^C for 16 h for CFU counting. For morphological observation, treated MRSA were fixed in 2.5% glutaraldehyde overnight at 4^o^C, washed with PBS (pH 7.4), dehydrated through graded ethanol (30%-100%), dried by critical point drying, coated with platinum, and observed by SEM.

**1.6 Live/Dead Bacterial Staining**

To assess the viability of MRSA after FeCo-N-DAC treatment, dual staining with acridine orange (AO) and propidium iodide (PI) was performed, followed by confocal laser scanning microscopy (CLSM). Log-phase MRSA was resuspended in PBS and adjusted to an OD_600_ of 0.5. The bacterial suspension was mixed with FeCo-N-DAC to a final concentration of 50 µg/mL in a 96-well plate and incubated at 37^o^C for 16 h. After incubation, AO and PI were added to each well at final concentrations of 20 µg/mL and 10 µg/mL, respectively. Samples were incubated in the dark for 30 min. Bacterial fluorescence images were acquired by CLSM. Live bacteria fluoresced green (AO), and dead bacteria fluoresced red (PI).

**1.7 Antibiofilm Activity of FeCo-N-DAC**

Sterile 6-well plates were each inoculated with 2.5 mL of log-phase MRSA suspension (1×10^7^ CFU/mL) and 5 mL of TSB medium. Plates were incubated at 37°C for 24 h to allow mature biofilm formation. After removing the supernatant, wells were washed three times with PBS to eliminate planktonic bacteria. Post-treatment, wells were washed and dried again, followed by staining with 5% (w/v) crystal violet for 20 min. After discarding the dye, plates were washed with deionized water until no excess dye remained. Then 33.3% (v/v) glacial acetic acid was added to dissolve the bound dye. The resulting solution was transferred to a 96-well plate, and absorbance at 590 nm was measured to quantify biofilm biomass.

**1.8 Safety Evaluation of FeCo-N-DAC**

*In vitro* hemolysis test: 300 µL fresh anticoagulated mouse whole blood was mixed with 300 µL PBS and different FeCo-N-DAC concentrations (12.5-200 µg/mL). After incubation at 37^o^C for 2 h, samples were centrifuged (8000 rpm, 5 min), and the absorbance of the supernatant was measured at 540 nm. Hemolysis (%) = [(As - An) / (Ap - An)] × 100%, where As = sample absorbance, An = PBS control, Ap = water control. *In vivo* hematology and biochemistry: Mice were divided into PBS and FeCo-N-DAC groups. Each mouse received a 50 µL intraperitoneal injection of PBS or FeCo-N-DAC (50 μg/mL). After 24 h, blood was collected from the orbital vein and analyzed for blood counts; the rest was centrifuged for serum biochemical analysis.

**1.9 Transcriptomic and Proteomic Analysis**

To explore sample similarity, Principal Component Analysis (PCA) was performed using the stats package in R software (version 4.4.1). Differential expression analysis was conducted using the limma package (version 3.60.4) in R software. The Benjamini-Hochberg method was applied to adjust P-values, reported as the False Discovery Rate (FDR). The criteria for defining differentially expressed molecules were as follows: For mouse skin tissue, differentially expressed mRNAs were defined as those with FDR < 0.01 and |Log_2_FC| > 2, while differentially expressed proteins were defined as those with FDR < 0.01 and |Log_2_FC| > 1. For porcine skin tissue, both differentially expressed mRNAs and proteins were defined as those with P-value < 0.05 and |Log_2_FC| > 0.6. To facilitate integrated analysis across transcriptomic and proteomic data, all molecules were represented using corresponding gene symbols. For functional enrichment analysis, Gene Ontology Biological Processes (GO-BP) and Kyoto Encyclopedia of Genes and Genomes (KEGG) pathway enrichment for the mouse skin tissue samples were performed using the Metascape online platform (version 3.5.20240901). For the porcine skin tissue samples, GO-BP and KEGG enrichment analyses were conducted using the ClueGO plugin (version 2.5.10) within Cytoscape software (version 3.10.0). Gene Set Enrichment Analysis (GSEA) was performed using the gseGO function from the R clusterProfiler package (version 4.12.6). To investigate protein-protein interactions, a Protein-protein interaction (PPI) network for the differentially expressed genes was constructed using the Search Tool for the Retrieval of Interacting Genes (STRING) database (version 12.0) and visualized using Cytoscape software. The MCODE plugin (version 2.0.2) for Cytoscape was used to identify densely connected network modules (core modules) and key genes within the PPI network. Concurrently, the CytoNCA plugin (version 2.1.6) was employed to calculate degree centrality (unweighted) for network nodes. Statistical analysis and visualization of all data were performed using GraphPad Prism statistical software (version 10.0), R software, and Cytoscape software (version 3.10.0).

**2. Supplementary Materials**

**Figure S1.** TEM image of FeCo-N-DAC.

**Figure S2.** (a, b) AFM image of FeCo-N-DAC. (c) The thickness distribution of FeCo-N-DAC as measured by the AFM image is shown in the yellow line.

**Figure S3.** The AC-HAADF-STEM images of FeCo-N-DAC from different regions.

**Figure S4.** (a) TEM and (b-h) the corresponding EDS element mapping of FeCo-N-DAC showing the presence of Fe, Co, C, N, and O.

**Figure S5.** The location of the EDS line scan of FeCo-N-DAC in Figure S4b is shown in the red arrow.

**Figure S6.** X-ray energy dispersive spectrum of FeCo-N-DAC confirmed the coexistence of Fe, Co, C, N, and O.

**Figure S7.** XRD patterns of FeCo-N-DAC.

**Figure S8.** Raman spectra of FeCo-N-DAC.

**Figure S9.** EPR spectra of FeCo-N-DAC.

**Figure S10.** (a) XPS survey spectra of FeCo-N-DAC. (b) High-resolution XPS for C 1s.

**Figure S11.** (a) Fe foil FT-EXAFS fitting curves of FeCo-N-DAC. (b) FT-EXAFS fitting of Fe foil for the FeCo-N-DAC at R space. (c) Co foil FT-EXAFS fitting curves of FeCo-N-DAC. (d) FT-EXAFS fitting of Co foil for the FeCo-N-DAC at R space.

**Figure S12.** WT-EXAFS plots of Co *K*-edge at R space form Co_3_O_4_.

**Figure S13.** UV-vis spectra of FeCo-N-DAC.

**Figure S14.** (a) Heating curve under 1064 nm laser irradiation (1.0 W/cm^2^) and cooling curve under room temperature of FeCo-N-DAC. (b) Linear fitting plots of time versus -Ln (*θ*) during the cooling period.

**Figure S15.** Relative catalytic activity of FeCo-N-DAC at different pH values. Inset digital photos: color change of corresponding samples.

**Figure S16.** Time-dependent absorption changes of TMB in the absence (a) and presence (b) of 1064 nm laser irradiation (1.0 W/cm^2^). UV-vis absorption spectra of OPD under the different concentrations of FeCo-N-DAC in acid buffer solution (pH = 5.6) without (c) and with (d) 1064 nm laser irradiation (1.0 W/cm^2^). Time-dependent absorption changes of OPD in the absence (e) and presence (f) of 1064 nm laser irradiation (1.0 W/cm^2^).

**Figure S17.** (a) UV-vis absorption spectra of TMB in the presence of FeCo-N-DAC under different concentrations of H_2_O_2_ at 25^o^C. (b) Lineweaver-Burk fitting for FeCo-N-DAC with H_2_O_2_ as substrates at 25^o^C. (c) UV-vis absorption spectra of TMB in the presence of FeCo-N-DAC under different concentrations of H_2_O_2_ at 50^o^C. (b) Lineweaver-Burk fitting for FeCo-N-DAC with H_2_O_2_ as substrates at 50 ^o^C.

**Figure S18.** DFT simulates the optimal structures (Left) and two plots of charge density differences of FeCo-N-DAC: top view of the 3D plot (Middle) and 2D display (Right).

**Figure S19.** MRSA inhibition effects of (a) H_2_O_2_ (0-0.8 mM) and (b) FeCo-N-DAC (0-400 µg/mL) at different concentrations.

**Figure S20.** The evolution of root mean square deviation of the membrane in control and FeCo-N-DAC group.

**Figure S21.** 2D graph of density evolution of membrane after 100 ns.

**Figure S22.** The abscess size quantitative statistics at different times under different treatments.

**Figure S23.** Quantitative analysis of the bacterial colonies from bacteria-infected tissues by different treatments on the 10th day.

**Figure S24.** Histological analysis of MRSA-infected tissues after undergoing different treatments. Histological images with H&E and Gram staining of the infected tissue were collected after a Day 10 treatment.

**Figure S25.** Quantitative measurements of (a) H&E, (b) Masson, and (c) Gram staining. I: Control; II: FeCo-N-DAC; III: FeCo-N-DAC+NIR-II; IV: FeCo-N-DAC+H_2_O_2_; V: FeCo-N-DAC+NIR-II+H_2_O_2_.

**Figure S26.** Representative histological images of IHC staining of Arg1 and iNOS.

**Figure S27.** Quantitative measurements of IHC staining of (a) Arg1 and (b) iNOS.


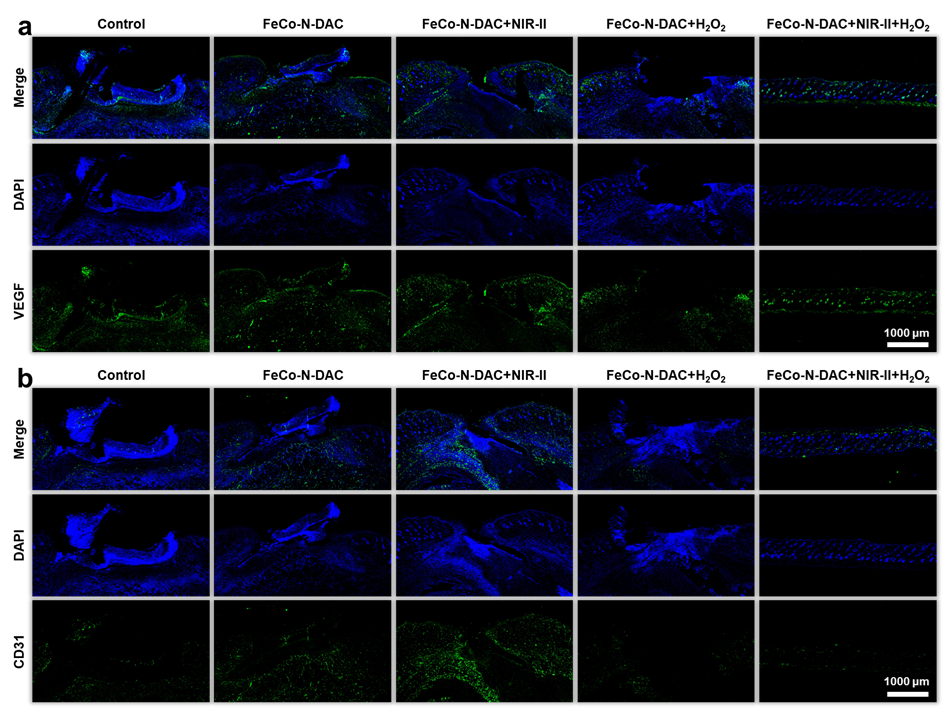


**Figure S28.** Representative images of VEGF and CD31 immunoﬂuorescence on the 10th day.

**Figure S29.** Heat map of the expression intensity of 14594 mRNA (left) and 7072 proteins (right) in mouse skin.

**Figure S30.** Visualization of GSEA BP results in mouse skin (a) TOP20 results of two omics; (b) "Fibrinolysis" in I*vs.*N proteomics. (c) "Fibrinolysis" in T*vs.*I proteomics.

**Figure S31.** The functional enrichment results of the transcriptomics and proteomics common differentially expressed genes.

**Figure S32.** Visualization of the functional enrichment results of genes that were up-regulated after infection and down-regulated after treatment in both omics. a) BP; b) KEGG.

**Figure S33.** Extraction of protein-protein interaction network and central network diagrams.


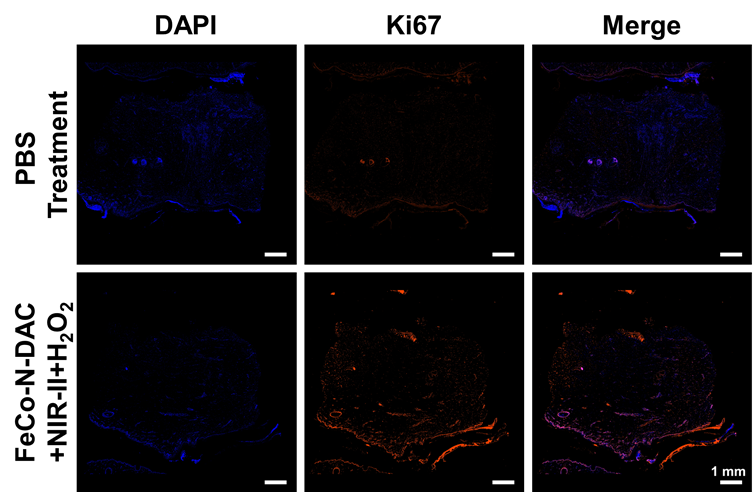


**Figure S34.** Representative images of Ki67 immunoﬂuorescence on the 30th day.

**Figure S35.** A heat map of the expression intensity of 14143 mRNA (left) and 5841 proteins (right) in pig skin.

**Figure S36.** Visualization of the TOP20 GSEA BP results of pig skin.

**Figure S37.** The functional enrichment results of genes in the I*vs.*N central network diagram: (a) mRNA, (b) protein.

**Figure S38.** Relative viabilities of L929 cells line after incubation with different concentrations of FeCo-N-DAC (0-100 μg/mL) for 24 h.

**Figure S39.** Biosafety assessments of FeCo-N-DAC. (a-d) Blood biochemistry and (e-k) blood routine evaluation of healthy mice hypodermic injection with 50 μL of FeCo-N-DAC suspension (50 μg/mL) or PBS at the 24th hour.

**Figure S40.** Illustrations of hemolysis activity of FeCo-N-DAC suspension of varying concentrations (0-200 μg/mL) and quantitative analysis.

**Table S1.** The common differential genes of the two omics and their intersection situations.

| Gene Name | Up-regulated in Ivs.N | Down-regulated in Ivs.N | Up-regulated in Tvs.I | Down-regulated in Tvs.I |
| --- | --- | --- | --- | --- |
| Acod1 | 1 | 0 | 0 | 0 |
| Cxcr2 | 1 | 0 | 0 | 1 |
| Krt6b | 1 | 0 | 0 | 0 |
| Fpr2 | 1 | 0 | 0 | 0 |
| Chil3 | 1 | 0 | 0 | 1 |
| Fpr1 | 1 | 0 | 0 | 1 |
| Ifitm1 | 1 | 0 | 0 | 1 |
| S100a9 | 1 | 0 | 0 | 1 |
| Hdc | 1 | 0 | 0 | 1 |
| Saa3 | 1 | 0 | 0 | 1 |
| Cd300lf | 1 | 0 | 0 | 1 |
| Arg1 | 1 | 0 | 0 | 0 |
| Srgn | 1 | 0 | 0 | 1 |
| Il1b | 1 | 0 | 0 | 1 |
| Msr1 | 1 | 0 | 0 | 1 |
| Fgr | 1 | 0 | 0 | 0 |
| Fcgr4 | 1 | 0 | 0 | 0 |
| Krt16 | 1 | 0 | 0 | 0 |
| Oas3 | 1 | 0 | 0 | 0 |
| Plek | 1 | 0 | 0 | 0 |
| Lcn2 | 1 | 0 | 0 | 1 |
| Hmox1 | 1 | 0 | 0 | 1 |
| Cd14 | 1 | 0 | 0 | 1 |
| Samsn1 | 1 | 0 | 0 | 1 |
| Olfm4 | 1 | 0 | 0 | 1 |

**Table S2.** The intersection of I*vs.*N differentially expressed genes.

| Gene Name | Up-regulated in transcriptomics | Down-regulated in transcriptomics | Up-regulated in proteomics | Down-regulated in proteomics |
| --- | --- | --- | --- | --- |
| PRDM8 | 1 | 0 | 0 | 0 |
| MGP | 1 | 0 | 0 | 0 |
| FAM198A | 1 | 0 | 0 | 0 |
| IL10 | 1 | 0 | 0 | 0 |
| POMGNT2 | 1 | 0 | 0 | 0 |
| RSPO1 | 1 | 0 | 0 | 0 |
| C5H12orf75 | 1 | 0 | 0 | 0 |
| RARRES2 | 1 | 0 | 0 | 0 |
| C1QTNF1 | 1 | 0 | 0 | 0 |
| DDAH1 | 1 | 0 | 0 | 0 |
| FAM20C | 1 | 0 | 0 | 0 |
| GADD45B | 1 | 0 | 0 | 0 |
| ASB9 | 1 | 0 | 0 | 0 |
| LOC100522011 | 1 | 0 | 0 | 0 |
| MSC | 1 | 0 | 0 | 0 |
| OLFML3 | 1 | 0 | 0 | 0 |
| WFDC1 | 1 | 0 | 0 | 0 |
| ZYX | 1 | 0 | 0 | 0 |
| NPAS2 | 1 | 0 | 0 | 0 |
| LTBR | 1 | 0 | 0 | 0 |
| FLNC | 1 | 0 | 1 | 0 |
| GZMK | 1 | 0 | 0 | 0 |
| HRH1 | 1 | 0 | 0 | 0 |
| C14H10orf128 | 1 | 0 | 0 | 0 |
| C1R | 1 | 0 | 0 | 0 |

**Table S3.** The intersection of T*vs.*I differentially expressed genes.

| Gene Name | Up-regulated in transcriptomics | Down-regulated in transcriptomics | Up-regulated in proteomics | Down-regulated in proteomics |
| --- | --- | --- | --- | --- |
| EGR1 | 1 | 0 | 0 | 0 |
| A2M | 1 | 0 | 0 | 0 |
| JPH4 | 1 | 0 | 0 | 0 |
| HBB | 1 | 0 | 0 | 0 |
| COX7A1 | 1 | 0 | 0 | 0 |
| F5 | 1 | 0 | 0 | 0 |
| PTCH2 | 1 | 0 | 0 | 0 |
| LOC106510300 | 1 | 0 | 0 | 0 |
| KHDRBS3 | 1 | 0 | 0 | 0 |
| DAPK1 | 1 | 0 | 0 | 0 |
| LOC100626407 | 1 | 0 | 0 | 0 |
| PDE1A | 1 | 0 | 0 | 0 |
| RGCC | 1 | 0 | 0 | 0 |
| VIPR2 | 1 | 0 | 0 | 0 |
| CXCL12 | 1 | 0 | 0 | 0 |
| DDIT4L | 1 | 0 | 0 | 0 |
| CLDN11 | 1 | 0 | 0 | 0 |
| TIMP4 | 1 | 0 | 0 | 0 |
| GABRE | 1 | 0 | 0 | 0 |
| LPIN1 | 1 | 0 | 0 | 0 |
| ID2 | 1 | 0 | 0 | 0 |
| ATF3 | 1 | 0 | 1 | 0 |
| CCL5 | 1 | 0 | 0 | 0 |
| RSPO4 | 0 | 1 | 0 | 0 |
| TCF19 | 0 | 1 | 0 | 0 |
| RSPO1 | 0 | 1 | 0 | 0 |
